# Discovery of *Schistosoma mekongi* circulating proteins and antigens in infected mouse sera

**DOI:** 10.1371/journal.pone.0275992

**Published:** 2022-10-13

**Authors:** Naphatsamon Uthailak, Poom Adisakwattana, Tipparat Thiangtrongjit, Yanin Limpanont, Phiraphol Chusongsang, Yupa Chusongsang, Kanthi Tanasarnprasert, Onrapak Reamtong

**Affiliations:** 1 Department of Molecular Tropical Medicine and Genetics, Faculty of Tropical Medicine, Mahidol University, Bangkok, Thailand; 2 Department of Helminthology, Faculty of Tropical Medicine, Mahidol University, Bangkok, Thailand; 3 Department of Social and Environmental Medicine, Faculty of Tropical Medicine, Mahidol University, Bangkok, Thailand; Consejo Superior de Investigaciones Cientificas, SPAIN

## Abstract

Schistosomiasis is a neglected tropical disease caused by an infection of the parasitic flatworms schistosomes. *Schistosoma mekongi* is a restricted *Schistosoma* species found near the Mekong River, mainly in southern Laos and northern Cambodia. Because there is no vaccine or effective early diagnosis available for *S*. *mekongi*, additional biomarkers are required. In this study, serum biomarkers associated with *S*. *mekongi-*infected mice were identified at 14-, 28-, 42-, and 56-days post-infection. Circulating proteins and antigens of *S*. *mekongi* in mouse sera were analyzed using mass spectrometry-based proteomics. **Serine** protease inhibitors and macrophage erythroblast attacher were down-regulated in mouse sera at all infection timepoints. In addition, 54 circulating proteins and 55 antigens of *S*. *mekongi* were identified. Notable circulating proteins included kyphoscoliosis peptidase and putative tuberin, and antigens were detected at all four infection timepoints, particularly in the early stages (12 days). The putative tuberin sequence of *S*. *mekongi* was highly similar to homologs found in other members of the genus Schistosoma and less similar to human and murine sequences. Our study provided the identity of promising diagnostic biomarkers that could be applicable in early schistosomiasis diagnosis and vaccine development.

## Introduction

Human schistosomiasis is a tropical parasitic disease caused by infections by blood flukes of the genus *Schistosoma* [1]. Among human parasitic diseases in general, schistosomiasis is ranked the second most widespread with regards to the numbers of morbidities and mortalities [2]. Over 250 million people worldwide are infected with schistosomiasis, and it is especially prevalent in Africa. The estimated annual mortality and risk of infection are 280,000 and 732 million cases, respectively, worldwide [2–4]. Schistosomes infect humans and other mammalian hosts when direct contact is made with freshwater contaminated with cercariae, allowing the cercariae to penetrate the skin [5]. Currently, there are six common causative species of human schistosomiasis, including *S*. *haematobium*, *S*. *mansoni*, *S*. *japonicum*, *S*. *guineensis*, *S*. *intercalatum*, and *S*. *mekongi* [2, 6]. *S*. *mekongi* has a distribution restricted to the area along the Mekong river, particularly in southern Laos and northern Cambodia [7–9]. Over 140,000 people are estimated to be infected with *S*. *mekongi* overall in the two countries [10].

The course of schistosomal infections are classified into three general stages: acute, established active, and late chronic, depending on egg excretions and clinical symptoms [6]. Unfortunately, there is no commercially available schistosomiasis vaccine [11]. Reliable initial diagnosis and the early treatment can dramatically decrease the morbidity and mortality risk of this disease. Until now, four main diagnoses for schistosomal infections have been reported: parasitological diagnosis, immunological detection, DNA-based detection, and biomarker detection [12]. Parasitological diagnosis (the Kato–Katz method) refers to the detection of eggs in stool and urine samples using microscopy. Because schistosome eggs are laid approximately 4–12 weeks post-infection, this method is not sensitive enough for an early diagnosis of schistosomiasis [13]. Immunological methods with higher sensitivity are also available, including the detection of schistosome antigens/host antibodies in the host’s blood circulation. However, antigen detection may not be appropriate for light infections [6]. Furthermore, antibody detection cannot distinguish between active and inactive infections because the antibodies can remain detectable for several years post-treatment [14]. Although DNA-based methods are applicable for early diagnosis because of their high sensitivity, their reliability is limited for patients after chemotherapy. Furthermore, *S*. *japonicum* DNA was reported to be undetectable by conventional PCR and loop-mediated isothermal amplification (LAMP) at weeks 8 and 14 post-chemotherapy, respectively. Thus, the DNA-based method is not ideal for distinguishing among different stages of infection or monitoring medical treatment efficacy [13]. As opposed to the other diagnostics, protein biomarkers can be applied to the detection of schistosomiasis at all stages, acute, i.e., established active, late chronic, and post-chemotherapy. Therefore, the identification of reliable biomarkers is important for the development and optimization of new diagnostic approaches with high efficiency [15]. Recently, many research efforts have focused on the identification of biomarkers for *Schistosoma* spp., such as *S*. *haematobium* [16, 17], *S*. *mansoni* [18], and *S*. *japonicum* [19, 20], to develop diagnostic sensitivity and specificity. Additionally, biomarkers are beneficial for the development of preventive and curative medications [15]. However, specific biomarkers are imperative for the diagnosis of *Schistosoma*. Many false-positive cases of schistosome infection are reported because of the low diagnosis specificities among healthy adults, pregnant women, and patients with other urine infections and hematuria [21, 22]. Even though some proteomic information on *S*. *mekongi* is available, and there are several biomarker candidates [23–26], those antigens may not be present at levels that facilitate immunological application. Thus, the search for novel biomarkers of *S*. *mekongi* for diagnosis and vaccine development is still ongoing [11].

The identification of reliable biomarker proteins can be performed using mass-spectrometry-based proteomic approaches [27]. Proteomics is a powerful technology for the study of proteins, including their structure, function, interaction, and composition [28], and has been applied to drug discovery, vaccine development, and diagnostic biomarker research [27, 29, 30]. Proteomics analysis of *S*. *mekongi* eggs has been used successfully to reveal somatic and excretory-secretory proteins [23, 25]. Moreover, phosphoproteomes of adult *S*. *mekongi* worms [22], and the effect of the commercial drug Praziquantel, has also been explored using this technique [31]. Although these studies provided useful data that are applicable for drug and vaccine development, only a few have focused on the discovery of reliable biomarkers for early-stage schistosomiasis diagnosis.

Therefore, this study aimed to identify additional diagnostic biomarkers for the early detection of *S*. *mekongi* and quantify changes in mouse serum proteins after *S*. *mekongi* infection at different timepoints (14-, 28-, 42-, and 56-days post-infection). Using mass-spectrometry-based proteomics, we also identified circulating parasitic proteins and antigens in infected mouse sera. Our work has provided a dataset of proteins detectable in *S*. *mekongi*-infected mouse sera, including reliable biomarker proteins that might be applied for vaccine development and the early diagnosis of schistosomiasis.

## Materials and methods

### Animals

The animal experiments were approved by the Faculty of Tropical Medicine Animal Care and Use Committee (FTM-ACUC), Mahidol University (approval number 015/2021). The experiments were conducted in eight-week-old female ICR mice. All efforts were made to minimize the number of animals (n = 3) used for the reliable statistical analysis.

### Infection of mice with *S*. *mekongi*

The mice were maintained in the Animal Care Unit, Faculty of Tropical Medicine, Mahidol University. Eight-week-old female ICR mice were percutaneously infected using abdominal exposure with cercariae of *S*. *mekongi*. Briefly, microscopic-counted 30 cercariae were picked up using hairloop and then gently touched at the abdomen of mice. Nembutal® (Pentobarbital) was used as an anesthesia. The mice did not show any sign of illness during 56 days after infection. Animal health and behaviour were monitored twice a day. Blood was collected from the submandibular vein before (pre-infection) and at 14, 28, 42, and 56 days post-infection. The collection tubes were left at room temperature for 30 min to allow blood clotting. Sera were then harvested using centrifugation (2,000g) at 4°C for 10 min and stored at -20°C until use. Three biological replications were performed. After experiments at 56 days post-infection, mice were simultaneously euthanized using the CO_2_-compressed carbon dioxide gas in cylinders.

### Separation of protein from mouse serum

Mouse sera (30 μg) from different infection time points (14, 28, 42, and 56 days post-infection) were separated using 12% SDS-PAGE. Protein bands were stained with Coomassie Blue G. After de-staining, protein bands were cut into 12 small pieces for in-gel digestion.

### Extraction of *S*. *mekongi* circulatory antigen

The co-immunoprecipitation between host antibody and *S*. *mekongi* circulatory antigen was performed using protein A/G magnetic beads (Pierce^TM^, Thermo Scientific, Waltham, MA USA), following the manufacturer’ instructions. Briefly, magnetic beads (50 μL; 0.5 mg) was gently mixed with 150 μL of binding/washing buffer and the supernatant was then discarded. Pooled sera (10 μL) at different infection time points were diluted with binding/washing buffer (490 μL), and mixed with beads at room temperature for 1 h. The supernatant was then discarded and beads were washed twice with 500 μL of binding/washing buffer. The elution was performed by addition of elution buffer (50 μL; 0.1M glycine, pH 2.0) and incubated at room temperature for 10 min by shaking, follows by supernatant collection using centrifugation. Eluted proteins were separated in 12% SDS-PAGE gel, and protein bands were visualized by staining with Coomassie Blue G. After de-staining, protein bands from each sample were cut into 10 pieces for the trypsin digestion.

### In-gel digestion

Protein gels were incubated in 25 mM ammonium bicarbonate buffer with 50% acetonitrile to eliminate Coomassie dye. Both protein reduction and alkylation were performed using 4 mM dithiothreitol (Sigma-Aldrich, St. Louis, MO, USA) in 50 mM ammonium bicarbonate buffer and 250 mM iodoacetamide (Sigma-Aldrich, St. Louis, MO, USA), respectively. Gels were then dehydrated with 100% acetonitrile and supernatant were discarded. Proteins were digested with trypsin (10 ng; Sigma-Aldrich, St. Louis, MO, USA) dissolved in 200 μL of 50 mM ammonium bicarbonate buffer, containing 5% acetonitrile. Tryptic peptides were extracted from gel by incubation with 200 μL of acetonitrile for 20 min. Supernatant was collected, and dried using a centrifuge vacuum concentrator. Peptides were then dissolved in 0.1% v/v formic acid for mass-spectrometric analysis.

### Mass spectrometric analysis

Mixture of tryptic peptides were applied to a nano liquid chromatography system (Dionex UltiMate 3000, Surrey, UK). Peptides were separated using an Acclaim PepMap RSLC nanoviper analytical column (75 μm x 15 cm, C18, 2 μm particle size, 100°A pore size; Thermo Scientific, Waltham, MA, USA) at flow rate of 300 nL/min. Both 0.1% formic acid in water (A) and 80% acetonitrile in 0.1% formic acid (B) were used as a mobile phase. Peptides were eluted using a 30 min gradient from 4% to 50% B, and applied into a micrO-TOF-Q mass spectrometer (Bruker Daltonics, Bremen, Germany). Data of mass spectrometry (MS) and tandem mass spectrometry (MS/MS) covered m/z ranges of 400–2000 and 50–1500, respectively. The exponentially modified protein abundance index (emPAI) values is used for estimation of protein amount in the proteomics [32, 33]. A Mascot generic file (.mgf) was obtained using the DataAnalysis 3.4 software (Bruker Daltonics, Bremen, Germany). The mgf. files were merged and proteins were identified using a Mascot Daemon version 2.3.2 (Matrix Science, London, UK). The in-house sequence database of *S*. *mekongi* was used to identify circulating antigens and proteins in mouse serum. The Mascot search accepted up to 1 missed cleavage and 0.8 Da of a peptide tolerance for MS and MS/MS spectra. Cysteine carbamidomethylation and methionine oxidation were identified as variable modifications. The exponentially modified protein abundance index was used to determine the protein abundance, semi-quantitatively. Data were obtained using a volcano plot with the statistical significance (t-test, *p*< 0.05) as calculated by the Perseus software platform. Protein-protein interactions were analysed using the STRING database.

### Bioinformatics

Sequence alignment and identity calculation were performed using the Clustal Omega software. All sequences were obtained from the non-redundant protein sequence database of the NCBI. Signal peptides existed in identified proteins were predicted using the SignalP 5.0 server with a SignalP score greater than 0.9 [34]. The prediction of non-classical protein secretion was performed using the SecretomeP 2.0 server with a SecretomeP score greater than 0.6 in mammalian proteins [35].

## Results

### Proteomic analysis of mouse sera infected with *S*. *mekongi*

**Fig 1:**
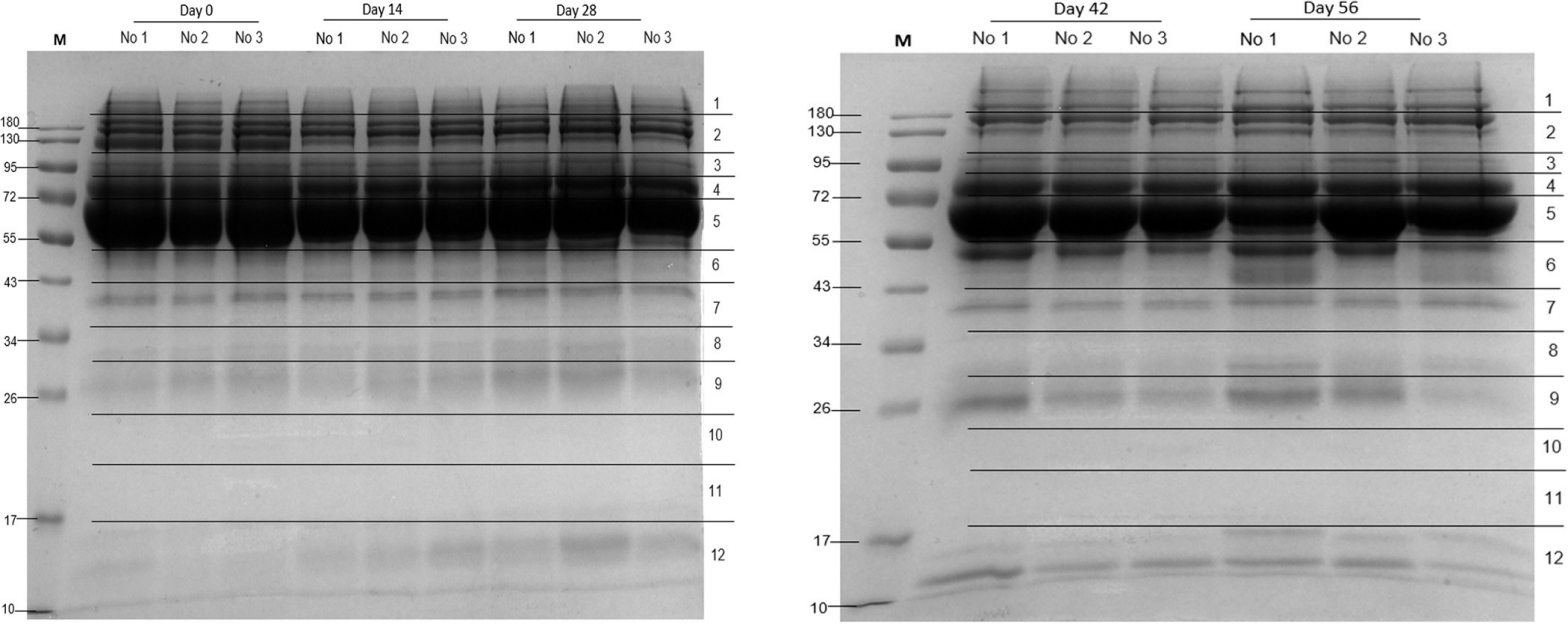
SDS-PAGE analysis of *S*. *mekongi*-infected mouse serum at 0- (pre-infection), 14-, 28-, 42-, and 56-days post-infection. M: marker; No. 1–3: mice number 1–3. The 12 horizontal sections represent excised protein bands used in mass spectrometric analysis.

Differential protein concentrations in mouse serum before infection and at 14-, 28-, 42, and 56-days post-infection were determined by separation with SDS-PAGE (Fig 1). Mass spectrometry analysis of the protein bands was used to identify the proteins (S1 Table).

**Fig 2:**
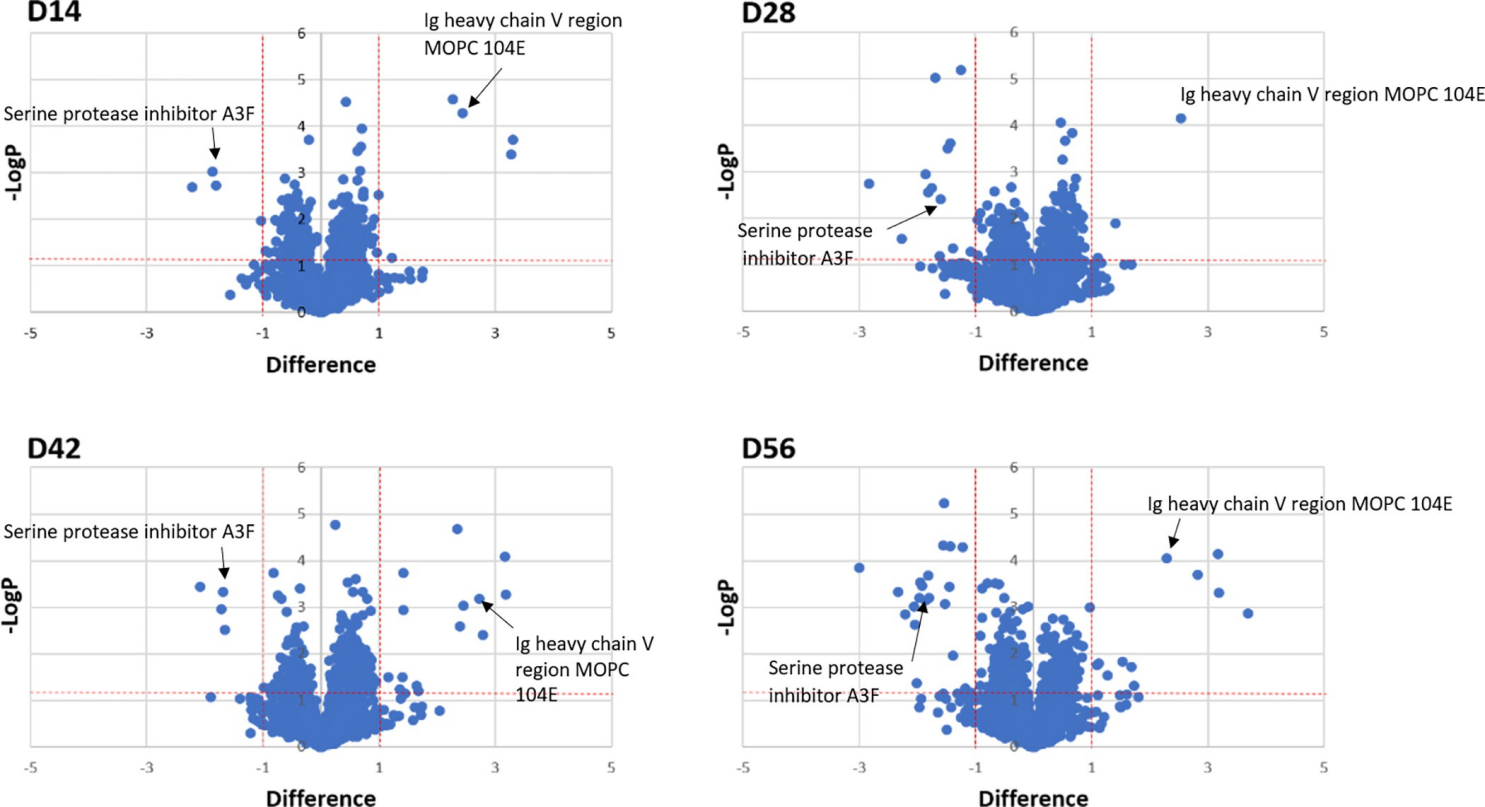
Comparison of protein expression in uninfected and infected mouse sera at 14- (D14), 28- (D28), 42- (D42), and 56- (D56) days post-infection with *S*. *mekongi* using volcano plots. Two vertical red lines represent differences as a minimum 2-fold changes relative to pre-infection conditions. The horizontal red line represents statistically significant at p-value <0.05. Dots above the horizontal red line with the difference more than 1 and less than -1 indicate up-regulated and down-regulated mouse serum proteins, respectively. Arrows indicate differential proteins found in all four post-infection time points.

**Fig 3:**
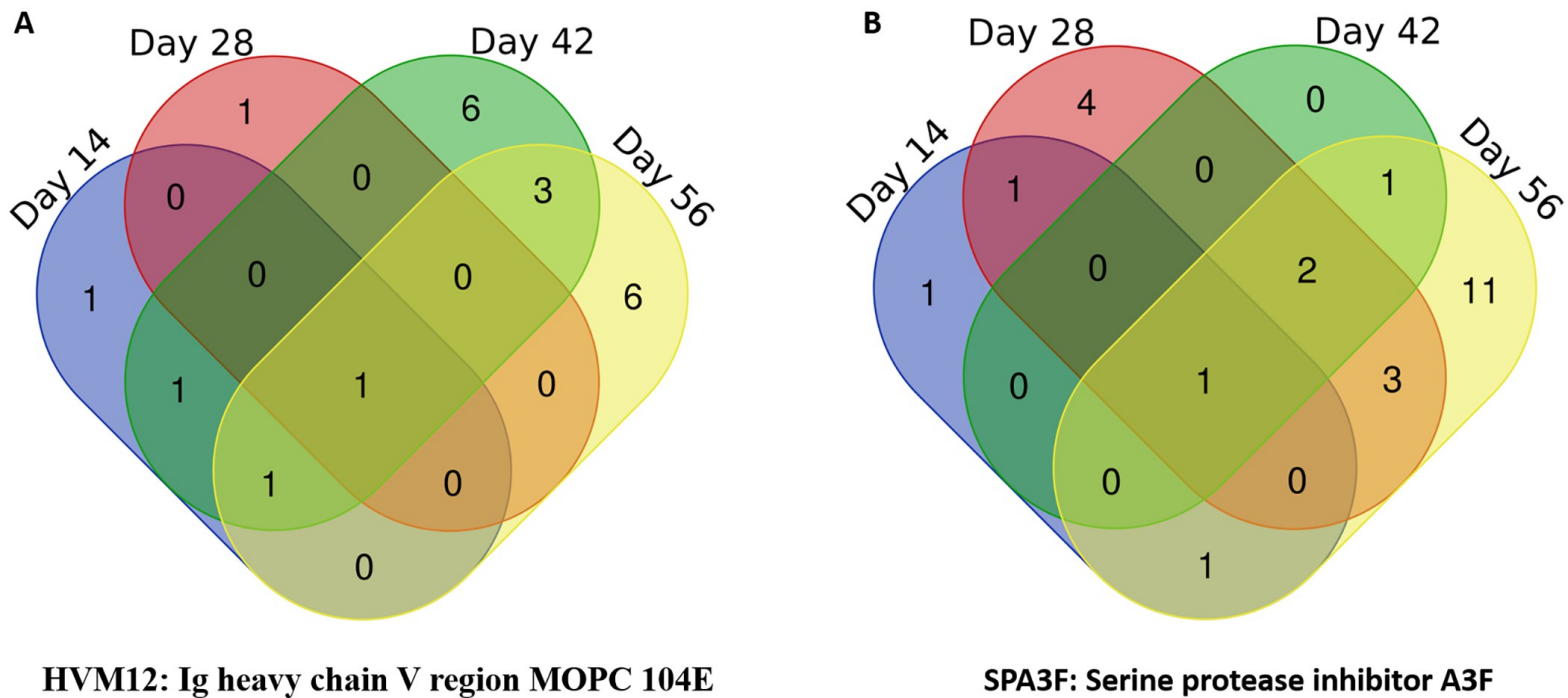
Up-regulated (A) and down-regulated (B) mouse serum proteins 14-, 28-, 42-, and 56-days post-infection with *S*. *mekongi*.

A comparison between the proteomes of uninfected and infected mouse sera was performed to determine both the up-regulation and down-regulation of protein expression levels. Many significantly differential mouse serum proteins were detected from all four post-infection timepoint samples (Fig 2). Overall, there was a higher number of down-regulated proteins than up-regulated proteins, i.e., 19 vs 24, respectively (Fig 3 and Table 1).

**Table 1:** Differential mouse serum proteins at 14-, 28-, 42-, and 56-days post-infection with *S*. *mekongi*. Up-regulated (bold) and down-regulated proteins were identified by a comparison between uninfected and infected mouse sera using LC-MS/MS and the UniProt protein database. *Mus musculus* was used as the taxonomy filter. Only significant differences detected in all three mice are shown in this table (p-value ≤ 0.05).

Accession	Protein	Score	Mass	Peptides	%cov	pI	Dif	-logP
**Day 14**								
**KV5A4**	**Ig kappa chain V-V region MOPC 149**	**164**	**12023**	**3**	**34.3**	**6.92**	**2.27**	**4.58**
**HVM12**	**Ig heavy chain V region MOPC 104E**	**125**	**12975**	**4**	**53.8**	**6.84**	**2.44**	**4.28**
**KV5AB**	**Ig kappa chain V-V region HP R16.7**	**243**	**11903**	**4**	**44.4**	**7.97**	**3.31**	**3.71**
**KV3AI**	**Ig kappa chain V-III region PC 6684**	**180**	**12032**	**4**	**34.2**	**7.98**	**3.27**	**3.39**
SPA3F	Serine protease inhibitor A3F	91	49952	6	17.8	4.79	-1.87	3.02
K1C19	Keratin, type I cytoskeletal 19	96	44515	3	9.7	5.28	-1.80	2.73
ACTS	Actin, alpha skeletal muscle	241	42024	6	34	5.23	-2.21	2.68
CP27B	25-hydroxyvitamin D-1 alpha hydroxylase,mitochondrial	39	56189	6	15	8.57	-1.03	1.97
**Day 28**								
**HVM12**	**Ig heavy chain V region MOPC 104E**	**125**	**12975**	**4**	**53.8**	**6.84**	**2.54**	**4.15**
**K2C6A**	**Keratin, type II cytoskeletal 6A**	**113**	**59299**	**5**	**13.4**	**8.04**	**1.42**	**1.89**
PPBT	Alkaline phosphatase, tissue-nonspecific isozyme	59	57419	5	14.7	6.42	-1.25	5.19
SPA3G	Serine protease inhibitor A3G	78	48990	7	21.8	6.06	-1.70	5.02
MAEA	Macrophage erythroblast attacher	69	45307	6	17.4	8.95	-1.43	3.62
K1C40	Keratin, type I cytoskeletal 40	67	48896	4	12.5	4.48	-1.47	3.50
ACTS	Actin, alpha skeletal muscle	241	42024	6	34	5.23	-1.86	2.96
KV3AJ	Ig kappa chain V-III region PC 7175	131	12003	4	34.2	7.01	-2.83	2.75
SPA3N	Serine protease inhibitor A3N	134	46688	6	19.1	5.59	-1.75	2.64
ACTC	Actin, alpha cardiac muscle 1	192	41992	6	27.6	5.23	-1.81	2.57
SPA3F	Serine protease inhibitor A3F	91	49952	6	17.8	4.79	-1.60	2.41
GPX3	Glutathione peroxidase 3	189	25409	6	37.2	8.33	-2.26	1.57
CO3	Complement C3	2149	186365	54	36.2	6.39	-1.38	1.36
**Day 42**								
**KV3AM**	**Ig kappa chain V-III region PC 2154**	**90**	**11692**	**2**	**27.8**	**5.83**	**2.35**	**4.67**
**KV3A7**	**Ig kappa chain V-III region TEPC 124**	**204**	**12331**	**4**	**35.7**	**10.02**	**3.17**	**4.09**
**MMP3**	**Stromelysin-1**	**58**	**53811**	**6**	**17.8**	**5.74**	**1.41**	**3.74**
**KV3AI**	**Ig kappa chain V-III region PC 6684**	**180**	**12032**	**4**	**34.2**	**7.98**	**3.18**	**3.28**
**HVM12**	**Ig heavy chain V region MOPC 104E**	**125**	**12975**	**4**	**53.8**	**6.84**	**2.73**	**3.18**
**KV3A4**	**Ig kappa chain V-III region 50S10.1**	**149**	**12035**	**3**	**44.1**	**4.9**	**2.45**	**3.04**
**NPL4**	**Nuclear protein localization protein 4 homolog**	**59**	**67974**	**5**	**13**	**6.01**	**1.42**	**2.94**
**KV5A4**	**Ig kappa chain V-V region MOPC 149**	**164**	**12023**	**3**	**34.3**	**6.92**	**2.39**	**2.59**
**KV5A3**	**Ig kappa chain V-V region K2 (Fragment)**	**238**	**12573**	**4**	**32.2**	**8.5**	**2.79**	**2.40**
**CSRN3**	**Cysteine/serine-rich nuclear protein 3**	**71**	**66080**	**8**	**15.6**	**4.65**	**1.15**	**1.49**
**CFAB**	**Complement factor B**	**765**	**84951**	**14**	**20.4**	**7.18**	**1.41**	**1.49**
**HVM63**	**Ig heavy chain Mem5 (Fragment)**	**144**	**25333**	**4**	**32.1**	**8.13**	**1.65**	**1.30**
SPA3N	Serine protease inhibitor A3N	134	46688	6	19.1	5.59	-2.07	3.44
SPA3F	Serine protease inhibitor A3F	91	49952	6	17.8	4.79	-1.69	3.33
MAEA	Macrophage erythroblast attacher	69	45307	6	17.4	8.95	-1.72	2.97
CS068	Uncharacterized protein C19orf68 homolog	45	50434	4	11	9.42	-1.65	2.51
**Day 56**								
**KV3A7**	**Ig kappa chain V-III region TEPC 124**	**204**	**12331**	**4**	**35.7**	**10.02**	**3.18**	**4.14**
**HVM12**	**Ig heavy chain V region MOPC 104E**	**125**	**12975**	**4**	**53.8**	**6.84**	**2.29**	**4.04**
**KV5A3**	**Ig kappa chain V-V region K2 (Fragment)**	**238**	**12573**	**4**	**32.2**	**8.5**	**2.83**	**3.70**
**KV3AI**	**Ig kappa chain V-III region PC 6684**	**180**	**12032**	**4**	**34.2**	**7.98**	**3.20**	**3.30**
**HPT**	**Haptoglobin**	**763**	**38727**	**18**	**49.3**	**5.88**	**3.69**	**2.86**
**ITIH3**	**Inter-alpha-trypsin inhibitor heavy chain H3**	**355**	**99304**	**9**	**12.1**	**5.7**	**1.54**	**1.82**
**CFAH**	**Complement factor H**	**1293**	**138992**	**36**	**34.5**	**6.6**	**1.12**	**1.80**
**CERU**	**Ceruloplasmin**	**1439**	**121074**	**36**	**32.1**	**5.53**	**1.09**	**1.75**
**CFAB**	**Complement factor B**	**765**	**84951**	**14**	**20.4**	**7.18**	**1.68**	**1.71**
**FXL12**	**F-box/LRR-repeat protein 12**	**42**	**37207**	**5**	**20.2**	**8.99**	**1.28**	**1.53**
**KV5A5**	**Ig kappa chain V-V region T1**	**54**	**14376**	**3**	**28.9**	**8.79**	**1.72**	**1.31**
K1H2	Keratin, type I cuticular Ha2	73	46360	4	13.3	4.75	-1.53	5.24
K1C40	Keratin, type I cytoskeletal 40	67	48896	4	12.5	4.48	-1.55	4.32
CS068	Uncharacterized protein C19orf68 homolog	45	50434	4	11	9.42	-1.43	4.31
MPP4	MAGUK p55 subfamily member 4	65	71955	7	18	5.31	-1.23	4.29
SAA4	Serum amyloid A-4 protein	130	15078	3	34.6	9.3	-3.00	3.84
SPA3G	Serine protease inhibitor A3G	78	48990	7	21.8	6.06	-1.81	3.68
KT33B	Keratin, type I cuticular Ha3-II	98	45834	8	25.2	4.79	-1.95	3.54
K1C18	Keratin, type I cytoskeletal 18	118	47509	6	15.8	5.22	-1.92	3.45
MAEA	Macrophage erythroblast attacher	69	45307	6	17.4	8.95	-1.46	3.44
K1C13	Keratin, type I cytoskeletal 13	171	47724	7	19.9	4.79	-2.33	3.33
K1C19	Keratin, type I cytoskeletal 19	96	44515	3	9.7	5.28	-1.96	3.20
KRT35	Keratin, type I cuticular Ha5	75	50497	6	15.6	4.9	-1.80	3.20
SPA3F	Serine protease inhibitor A3F	91	49952	6	17.8	4.79	-1.86	3.14
K1C15	Keratin, type I cytoskeletal 15	119	49107	8	21.9	4.79	-1.53	3.07
ACTC	Actin, alpha cardiac muscle 1	192	41992	6	27.6	5.23	-2.06	3.01
APOM	Apolipoprotein M	103	21259	5	24.7	6.08	-2.20	2.84
SPA3N	Serine protease inhibitor A3N	134	46688	6	19.1	5.59	-2.04	2.63
KRT36	Keratin, type I cuticular Ha6	90	52757	9	23.5	4.99	-1.39	1.96
HBE	Hemoglobin subunit epsilon-Y2	66	16126	3	29.9	7.9	-2.01	1.36

Fourteen-days post-infection, four mouse serum proteins were up-regulated (various Ig kappa and heavy chains) and four proteins were down-regulated (serine protease inhibitor A3F, keratin type I cytoskeletal 19, actin alpha skeletal muscle, and 25-hydroxyvitamin D-1 alpha hydroxylase). On day-28 post-infection, Ig heavy chain V region MOPC 104E and keratin type II cytoskeletal 6A were up-regulated, whereas 11 proteins were down-regulated in the infected mouse: alkaline phosphatase, serine protease inhibitors (A3G, A3N, and A3F), macrophage erythroblast attacher, keratin type I cytoskeletal 40, actins (alpha skeletal and cardiac muscle), Ig kappa chain, glutathione peroxidase 3, and complement C3. At 42-days post-infection, 12 infected mouse serum proteins were up-regulated, including various Ig kappa and heavy chains, stromelysin-1, nuclear protein localization protein 4, cysteine/serine-rich nuclear protein 3, and complement factor B. Four proteins were down-regulated (serine protease inhibitor and macrophage erythroblast attacher). At 56-days post-infection, 11 mouse serum proteins were up-regulated (various Ig kappa and heavy chains, haptoglobin, inter-alpha-trypsin inhibitor heavy chain H3, complement factor [H and B], ceruloplasmin, and F-box/LRR-repeat protein 12) and 19 were down-regulated (various keratin types, MAGUK p55 subfamily member 4, serum amyloid A-4 protein, serine protease inhibitors, macrophage erythroblast attacher, actin alpha cardiac muscle 1, apolipoprotein M, and hemoglobin subunit epsilon-Y2). The up-regulated and down-regulated mouse serum proteins significantly increased in a manner correlated with infection time. Although there was no discernable pattern to the protein changes, the highest number of both up-regulated and down-regulated proteins was observed at 56-days post-infection. Notably, one up-regulated protein (Ig heavy chain V region MOPC 104E) and one down-regulated protein (serine protease inhibitor A3F) were identified at all four infection timepoints. Therefore, these differential proteins might be useful when studying host responses against *S*. *mekongi* infection.

### Protein–protein interaction analysis of *S*. *mekongi*-infected mouse sera

**Fig 4:**
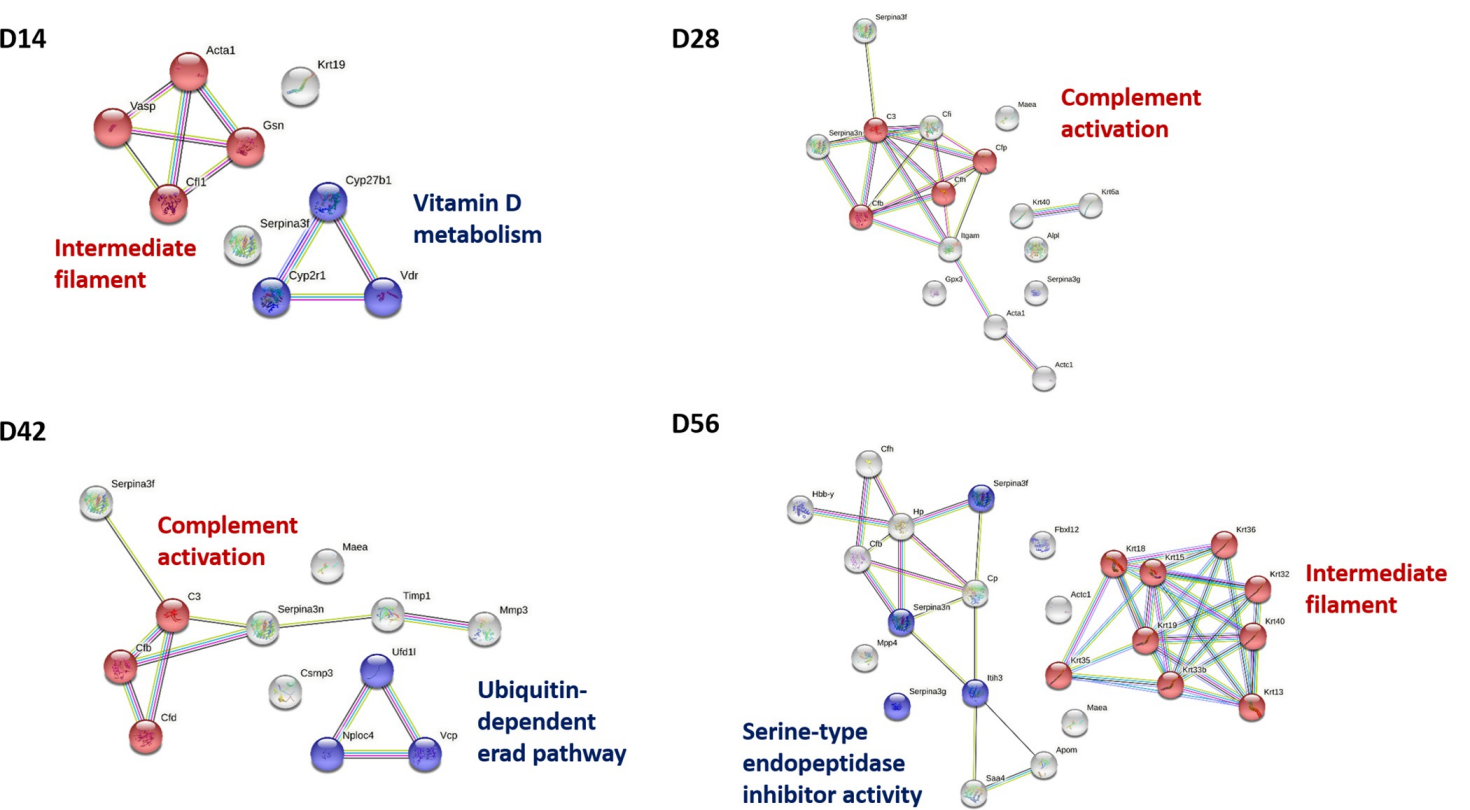
Protein-protein interactions of up-regulated and down-regulated *S*. *mekongi*-infected mouse serum proteins at 14-, 28-, 42-, and 56-days post-infection. The protein-protein interaction network was created using String database. Red and blue nodes indicate proteins in each pathway predicted to be altered with *S*. *mekongi* infection. Color nodes (red and blue) represent query proteins with first shell of interactors. White nodes represent proteins with second shell of interactors. Empty nodes indicate unknown 3D structural protein. Filled nodes indicate proteins with known or predicted 3D structure. Edges represent protein-protein associations with different types; red line: presence of fusion evidence; green line: neighborhood evidence; blue line: cooccurrence evidence; purple line: experimental evidence; yellow line: textmining evidence; light blue line: database evidence; black line: co-expression evidence. Acta1: actin alpha 1 skeletal muscle; Gsn: gelsolin; Cfl1: cofilin 1; Vasp: vasodilator stimulated phosphoprotein; Cyp27b1: cytochrome P450 family 27 subfamily B member 1; Cyp2r1: cytochrome P450 family 2 subfamily R member 1; Vdr: vitamin D receptor; C3: complement C3; Cfp: complement factor properdin; Cfh: complement factor H; Cfb: complement factor B; Cfd: complement factor D; Ufd1: ubiquitin recognition factor in ER associated degradation 1; Vcp: valosin containing protein; Nploc4: NPL4 homolog, ubiquitin recognition factor; Serpina3f: serpin clade A member 3F; Serpina3n: serpin clade A member 3N; Serpina3g: serpin clade A member 3G; Ithi3: inter-alpha-trypsin inhibitor heavy chain 3; Krt: keratin.

Protein–protein interactions in the infected mouse sera at different timepoints (14-, 28-, 42-, and 56-days post-infection) were analyzed using the STRING database (Fig 4), and all samples revealed differential molecular processes. Intermediate filament and vitamin D metabolism were differential pathways associated with *S*. *mekongi* infection on day 14, and complement activation was a differential pathway in mouse serum on day-28 post-infection. Additionally, ubiquitin-dependent and complement activation processes were predicted differential pathways in the 42-day post-infection serum, whereas day-56 mouse serum contained two differential pathways: intermediate filament and serine-type endopeptidase inhibitor activity. This work provides additional information on host responses during schistosomiasis.

### Detection of circulating *S*. *mekongi* proteins in infected-mouse sera

**Fig 5:**
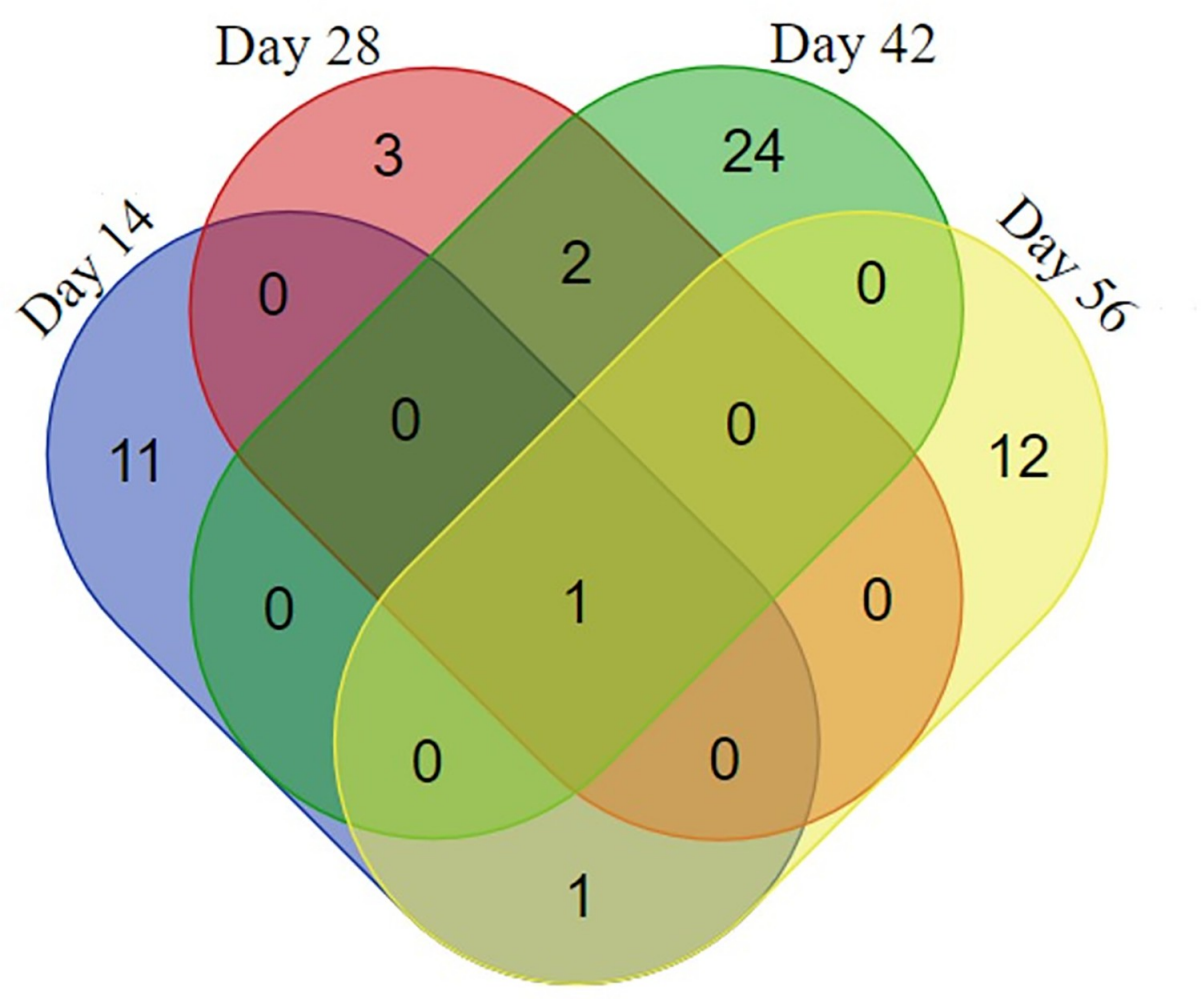
Circulating proteins of *S*. *mekongi* identified in infected mouse sera at 14-, 28-, 42-, and 56-days post-infection using mass-spectrometric analysis. Numbers represent the number of *S*. *mekongi* circulating proteins identified by mass spectrometry.

Fifty-four circulating proteins from *S*. *mekongi* were identified based on a proteomics approach (Table 2 and S2 Table). *S*. *mekongi* proteins were detected at all four infection timepoints (14-, 28-, 42-, and 56-days post-infection), whereas no *S*. *mekong*i proteins found in uninfected mouse sera. At 14-days post-infection, putative pre-mRNA-splicing factor ATP-dependent RNA helicase (Gene.11529::comp3258), kyphoscoliosis peptidase (Gene.7318::comp2030), and guanine nucleotide-releasing factor 2 (Gene.23156::comp7299) from the parasite were identified with high confidence. Putative DNA replication helicase DNA2 (Gene.8202::comp2307) and putative nephrin (Gene.21550::comp6617) had the highest scores in the 28-day-infected mouse sera. On day-42 post-infection, the parasite proteins DNA polymerase (Gene.25399::comp8617), putative nephrin, protein split ends (Gene.12563::comp3554), microtubule-associated serine/threonine-protein kinase 4 (Gene.7582::comp2109), neuropathy target esterase/swiss cheese-related protein (Gene.7383::comp2055), putative dynein heavy chain (Gene.30001::comp18484), and kyphoscoliosis peptidase were detected with the highest scores. Additionally, putative tensin (Gene.10459::comp2932), rootletin (Gene.26160::comp9193), putative helicase (Gene.4291::comp1166), and aminopeptidase N (Gene.20904::comp6346) were found with the highest confidence on day-56 post-infection. Importantly, only one protein was observed at all four infection timepoints, namely kyphoscoliosis peptidase (Fig 5). Kyphoscoliosis peptidase could not be observed in the uninfected mouse sera at any time (S3 Table); therefore, it may be an applicable biomarker for diagnosis in the early stages (2 weeks) and other diagnostic stages of *S*. *mekongi* infection.

**Table 2:** Circulating proteins of *S*. *mekongi* identified in infected mouse sera at 14-, 28-, 42-, and 56-days post-infection. LC-MS/MS and the in-house database were used, with *S*. *mekongi* set as the taxonomy filter. The SignalP score (>0.9) and Secretome score (>0.6) were used to define classical and non-classical protein secretion, respectively.

Accession	Protein	Score	Mass	Peptides	%cov	pI	Ref.
Day 14							
Gene.10399::comp2913	Uncharacterized protein	294	282914	12	7.6	7.83	-
Gene.11529::comp3258	Putative pre-mRNA-splicing factor ATP-dependent RNA helicase (EC 3.6.1.-)	221	86647	9	15.8	7.08	-
Gene.20085::comp5987	Uncharacterized protein	174	70000	7	12.8	6.79	-
Gene.22185::comp6852	Putative hepatoma derived growth factor	174	111836	7	9.7	6.55	-
Gene.22377::comp6941	Uncharacterized protein	93	77293	6	14.1	6.61	-
Gene.23156::comp7299	Guanine nucleotide-releasing factor 2	203	169577	9	9.6	6.35	-
Gene.26208::comp9251	Uncharacterized protein (Fragment)	130	26229	5	26.9	9.11	-
Gene.2677::comp680	Uncharacterized protein	180	88517	10	20.6	6.14	-
Gene.26963::comp10046	Putative erythrocyte membrane protein	195	189608	8	8.1	5.45	-
Gene.27657::comp11049	Uncharacterized protein	96	136193	3	2.3	5.94	-
Gene.28495::comp12673	FMRFamide receptor	92	55904	3	4.8	9.23	[36]
Gene.29504::comp15887	Uncharacterized protein	178	117178	12	14.2	6.63	-
Gene.7318::comp2030	Kyphoscoliosis peptidase	211	177264	9	9	6.29	[37]
Day 28							
Gene.15005::comp4331	Eukaryotic translation initiation factor 2	171	80568	7	16.2	8.52	[36]
Gene.21550::comp6617	Putative nephrin	191	177909	8	6.8	7.57	[36]
Gene.23554::comp7498	Uncharacterized protein	157	195126	6	4.9	6.8	-
Gene.27713::comp11132	Uncharacterized protein CXorf22	261	244776	14	8.9	7.11	-
Gene.8202::comp2307	Putative dna replication helicase dna2	271	166438	12	11	8.03	-
Gene.7318::comp2030	Kyphoscoliosis peptidase	174	177264	7	6.8	6.29	[37]
Day 42							
Gene.12563::comp3554	Protein split ends	296	283955	14	7.3	9.02	[36]
Gene.12644::comp3565	Uncharacterized protein	190	95572	8	11.6	5.87	-
Gene.13033::comp3680	Zinc finger MYND domain-containing protein 11	182	129919	9	12	8.95	-
Gene.14322::comp4118	Uncharacterized protein (Fragment)	225	245658	13	9.5	6.94	-
Gene.15408::comp4438	Uncharacterized protein	110	39582	5	15.5	6.79	-
Gene.16168::comp4709	Putative ubiquitin-protein ligase	211	133726	9	10	8.72	-
Gene.16334::comp4756	Putative polybromo-1	214	235501	9	6.9	6.68	[36]
Gene.18895::comp5607	DNA helicase (EC 3.6.4.12)	236	100413	10	12.2	6.51	-
Gene.19132::comp5686	Voltage-dependent calcium channel	238	143033	10	11.1	6.78	-
Gene.19910::comp5936	Niemann-Pick C1 protein	137	122638	5	7.5	6.41	-
Gene.20208::comp6064	Uncharacterized protein	236	113015	10	15.9	7.32	-
Gene.20548::comp6198	Putative otopetrin	131	76266	5	10.8	8.68	-
Gene.20727::comp6262	Intron-binding protein aquarius	186	186768	10	9.1	6.05	-
Gene.20796::comp6283	Centrosomal protein of 135 kDa	211	103964	8	10.6	6.05	[36]
Gene.21550::comp6617	Putative nephrin	313	177909	12	9.4	7.57	[36]
Gene.23824::comp7615	GDNF-inducible zinc finger protein 1	185	132913	10	10.3	7.33	-
Gene.25399::comp8617	DNA polymerase (EC 2.7.7.7)	330	248957	14	10.6	6.14	-
Gene.27713::comp11132	Uncharacterized protein CXorf22	236	244776	13	9.5	7.11	-
Gene.28162::comp11926	Uncharacterized protein (Fragment)	155	116439	8	9.3	8.85	-
Gene.30001::comp18484	Putative dynein heavy chain	274	208883	11	9.1	5.73	[36]
Gene.4085::comp1122	Uncharacterized protein	283	127610	12	14.9	6.54	-
Gene.6274::comp1757	NADH dehydrogenase (Ubiquinone) Fe-S protein 2	136	56398	5	16.3	8.69	-
Gene.7318::comp2030	Kyphoscoliosis peptidase	263	177264	10	7.7	6.29	[37]
Gene.7383::comp2055	Neuropathy target esterase/swiss cheese-related protein	276	196893	12	9	8.15	*-*
Gene.7582::comp2109	Microtubule-associated serine/threonine-protein kinase 4	295	279663	13	8.1	7.78	*-*
Gene.8602::comp2420	Type II inositol 1,4,5-trisphosphate 5-phosphatase	140	135075	10	13.2	6.58	-
Gene.9495::comp2699	Uncharacterized protein	246	224841	12	9.2	9.21	-
Day 56							
Gene.10459::comp2932	Putative tensin	257	214499	11	8.8	8.78	-
Gene.14384::comp4145	Putative homeodomain transcription factor 2 (Fragment)	231	180240	10	9.8	8.68	-
Gene.1799::comp453	Translation initiation factor IF-2 unclassified subunit	113	71016	4	6.4	6.23	-
Gene.20085::comp5987	Uncharacterized protein	143	70000	6	14	6.79	-
Gene.20229::comp6072	Putative stromal antigen	197	151673	10	11.1	5.99	-
Gene.20904::comp6346	Aminopeptidase N	247	91200	10	16.7	6.39	[36, 37]
Gene.21793::comp6693	Serine/threonine-protein phosphatase (EC 3.1.3.16)	130	75414	6	11.1	6.22	[36]
Gene.22888::comp7169	Uncharacterized protein	222	146198	10	11.7	6.06	-
Gene.26160::comp9193	Rootletin	251	278235	18	8.7	5.66	[36]
Gene.4291::comp1166	Helicase, putative	249	183327	13	12.2	6.09	-
Gene.7318::comp2030	Kyphoscoliosis peptidase	191	177264	13	10.1	6.29	[37]
Gene.7365::comp2049	Tensin-3	201	134542	9	12.6	9.24	-
Gene.7749::comp2156	WD repeat protein 57	137	40426	5	19.6	7.6	-
Gene.8969::comp2536	Uncharacterized protein	271	332314	12	6.3	6.75	-

*Ref: Secreted *S*. *mekongi* proteins identified in previous study

### Determination of circulating *S*. *mekongi* antigens in mouse antigen–antibody complexes

**Fig 6:**
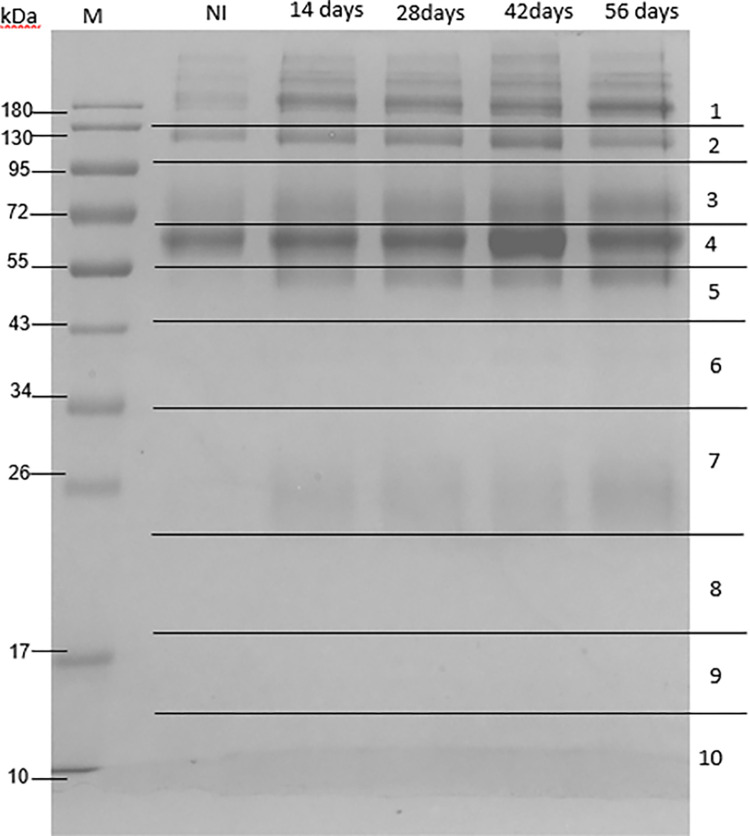
SDS-PAGE analysis of circulating antigens from immune complexes in sera from mice with and without *S*. *mekongi* infection. Immune complexes were enriched using protein A/G magnetic beads and separated with 12% SDS-PAGE. M: Marker; NI: Uninfected; 14-, 28-, 42-, and 56-days post-infection. The 10 horizontal sections represent regions excised for mass spectrometric analysis.

**Fig 7:**
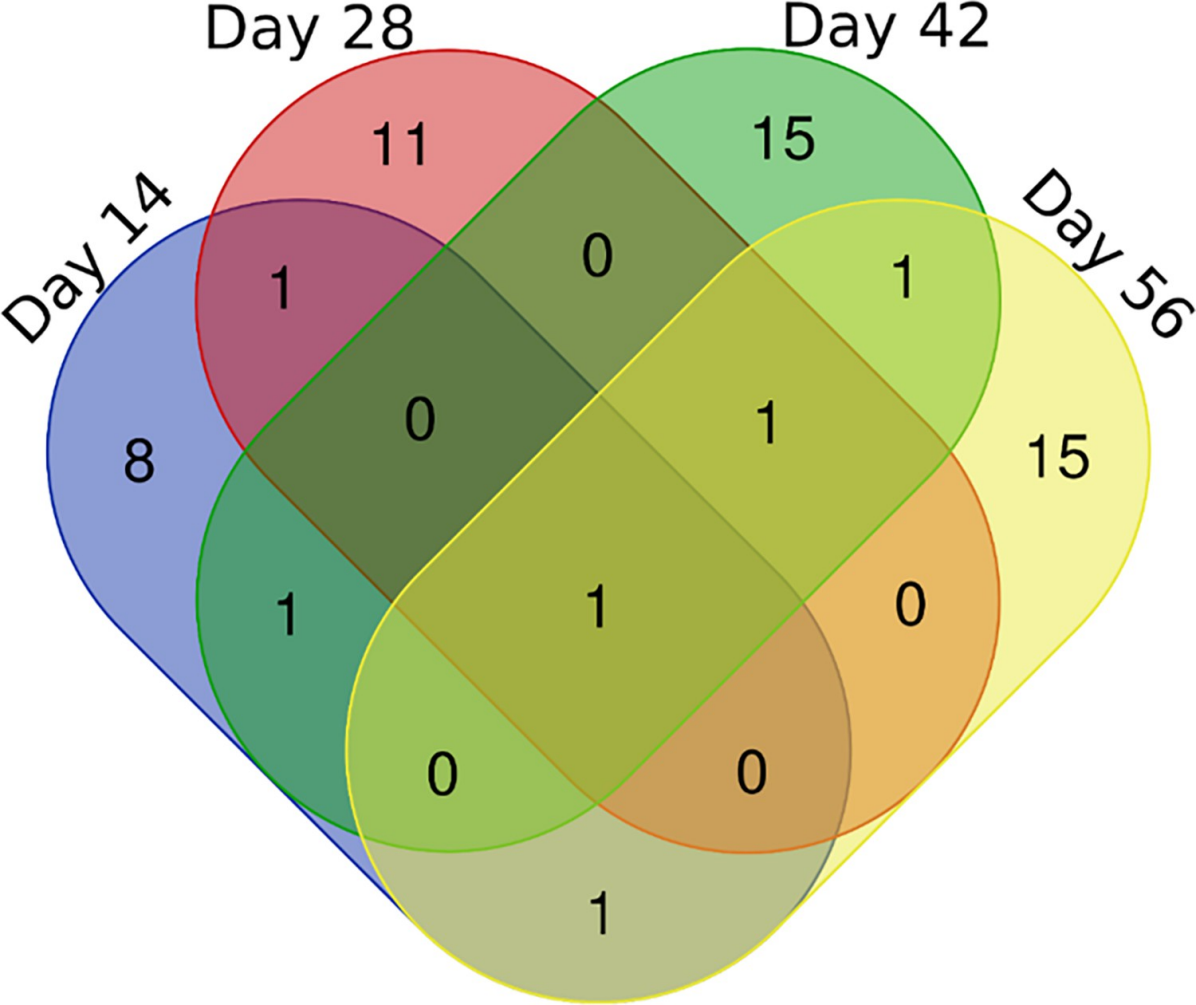
Circulating antigens of *S*. *mekongi* identified in infected mouse sera at 14-, 28-, 42-, and 56-days post-infection using mass spectrometric analysis. Numbers represent number of *S*. *mekongi* circulating antigens identified by mass spectrometry.

Immune complexes in mouse sera were separated and observed using SDS-PAGE (Fig 6). Analysis of uninfected mouse sera revealed protein bands corresponding to common antibodies in mouse blood. Compared with uninfected sera, the infected sera showed 55 and 26 kDa protein bands of significantly increased intensity. Thus, gels 5 and 7 were analyzed and identified using mass spectrometry and the UniPlot protein database. As expected, no *S*. *mekongi* proteins were detected in the immune complexes of the uninfected mouse sera. Overall, 55 circulating *S*. *mekongi* antigens were identified from immune complexes of infected mouse sera (Fig 7, Table 3 and S4 Table), in which the numbers of *S*. *mekongi* antigens identified at 14-, 28-, 42-, and 56-days post-infection were 12, 14, 19, and 19, respectively. Notably, putative tuberin (Gene.10133::comp2839) was detected at all four post-infection timepoints, whereas tubulin tyrosine ligase-related protein (Gene.29353::comp15247) and suppressor of cytokine signaling 7 (Gene.22611::comp7045) were identified in the early stages of infection (14 days). Therefore, these three identified proteins might be useful for the development of diagnostic tests sensitive to the early stages.

**Table 3:** Identification of circulating *S*. *mekongi* antigens from infected mouse immune complexes at 14-, 28-, 42-, and 56-days post-infection. *S*. *mekongi* proteins were identified using LC-MS/MS and the in-house database, with *S*. *mekongi* set as the taxonomy filter.

Accession	Protein	Score	Mass	Peptides	%cov	pI
**Day 14**						
Gene.29853::comp17559	Uncharacterized protein	36	11834	1	8.7	9.67
Gene.6613::comp1814	DNA-directed RNA polymerase subunit	56	12712	2	29.9	8.29
Gene.28118::comp11837	Putative vasohibin	43	24891	1	7.4	10.25
Gene.29887::comp17726	Uncharacterized protein	38	41000	1	2.8	8.47
Gene.17102::comp4985	Putative zinc finger protein	43	46833	1	5.6	8.77
Gene.148::comp23	Uncharacterized protein	58	46501	2	8	9.76
Gene.4692::comp1288	SJCHGC07480 protein (Fragment)	45	51777	1	4.7	7.23
Gene.29353::comp15247	Tubulin tyrosine ligase-related	45	123189	1	1.5	8.72
Gene.22611::comp7045	Suppressor of cytokine signaling 7	99	111285	4	6.7	8.86
Gene.10470::comp2937	WD repeat-containing protein 26	118	109261	5	8.9	5.7
Gene.10133::comp2839	Putative tuberin	125	242948	5	3.9	6.03
Gene.15811::comp4573	HEAT repeat-containing protein 1	222	251512	10	6.8	6.9
**Day 28**						
Gene.391::comp63	Ribosomal protein L26 (SJCHGC01959 protein)	38	16669	1	7	10.74
Gene.31564::comp30758	Endonuclease-reverse transcriptase	43	22141	1	6.9	6.04
Gene.4251::comp1155	Px19-like protein	36	24482	1	4.7	9.64
Gene.27438::comp10660	Uncharacterized protein	80	37875	3	13	5.78
Gene.4692::comp1288	SJCHGC07480 protein (Fragment)	42	51777	1	4.7	7.23
Gene.25955::comp9018	Uncharacterized protein	36	52664	1	2	8.43
Gene.7015::comp1924	Guanine-nucleotide-exchange-factor, putative	44	71738	1	4.1	9.39
Gene.13969::comp4009	Uncharacterized protein	42	80571	1	2	7.89
Gene.27309::comp10463	Uncharacterized protein	61	92839	2	3.9	8.63
Gene.17389::comp5102	Uncharacterized protein	100	78741	4	11.1	7.41
Gene.10078::comp2826	Transducin-like enhancer protein 3	136	102178	6	5.6	6.64
Gene.7023::comp1924	Ras-specific guanine nucleotide-releasing factor RalGPS2	64	157220	2	3.4	9.09
Gene.23237::comp7329	Protein kinase	76	172673	3	2.1	9.21
Gene.10133::comp2839	Putative tuberin	88	242948	3	2.5	6.03
**Day 42**						
Gene.29853::comp17559	Uncharacterized protein	37	11834	1	8.7	9.67
Gene.6609::comp1814	DNA-directed RNA polymerase subunit	37	13384	1	7.8	5.81
Gene.391::comp63	Ribosomal protein L26 (SJCHGC01959 protein)	38	16669	1	7	10.74
Gene.6633::comp1824	SJCHGC05758 protein	57	23991	2	4.9	8.63
Gene.3240::comp844	Serine/threonine-protein phosphatase (EC 3.1.3.16)	64	37322	2	10.7	6.34
Gene.24866::comp8236	Uncharacterized protein	62	44698	2	11.5	6.89
Gene.2701::comp687	Putative slit-robo rho gtpase activating protein	37	57525	1	2.5	4.92
Gene.29214::comp14782	p2X purinoceptor 4	37	52483	1	2	5.48
Gene.29102::comp14476	Cystic fibrosis transmembrane conductance regulator	62	64435	2	5.7	9.87
Gene.2210::comp533	Cullin 3	57	63525	2	4.6	6.54
Gene.9306::comp2645	Tyrosine-protein phosphatase non-receptor type 11	65	87197	2	4.7	7.02
Gene.17809::comp5238	Mername-AA248 (C02 family)	63	78103	2	6.8	6.16
Gene.26966::comp10053	Helicase POLQ-like	97	94383	4	5.6	6.96
Gene.29213::comp14782	p2X purinoceptor 4	57	101436	2	2.6	8.85
Gene.22090::comp6811	Uncharacterized protein	79	142820	3	3.1	6.94
Gene.19940::comp5946	Protein Smaug 2	77	168752	3	2.6	6.05
Gene.10133::comp2839	Putative tuberin	110	242948	4	4.2	6.03
Gene.23196::comp7320	Acetyl-CoA carboxylase / biotin carboxylase (Fragment)	220	302581	10	6.4	6.33
Gene.3614::comp956	Subfamily M23B non-peptidase homologue (M23 family)	118	242425	5	3.8	6.39
**Day 56**						
Gene.33195::comp60874	Alpha-2-macroglobulin-like protein 1	54	31684	4	23.5	5.91
Gene.23907::comp7644	Uncharacterized protein	39	15830	1	6.8	7.12
Gene.391::comp63	Ribosomal protein L26 (SJCHGC01959 protein)	56	16669	1	7	10.74
Gene.29708::comp16756	SJCHGC02480 protein (Fragment)	16	16797	2	21.5	8.86
Gene.34419::comp125314	Uncharacterized protein	37	23344	2	17.8	8.99
Gene.3059::comp801	Uncharacterized protein	17	30597	1	3.6	6.23
Gene.26154::comp9180	Uncharacterized protein	27	36988	1	3.1	9.2
Gene.30784::comp23275	Uncharacterized protein	17	40681	2	6.3	9.11
Gene.24745::comp8149	5-hydroxytryptamine receptor 1	21	47002	1	2.4	8.81
Gene.21224::comp6490	Transcription initiation factor tfiid 55 kD subunit-related	30	61621	1	2.5	4.7
Gene.9306::comp2645	Tyrosine-protein phosphatase non-receptor type 11	23	87197	2	4.4	7.02
Gene.29353::comp15247	Tubulin tyrosine ligase-related	23	123189	4	7.4	8.72
Gene.9257::comp2626	Nuclear cap-binding protein subunit 1	17	115297	3	3.9	5.47
Gene.23242::comp7333	Aminopeptidase (EC 3.4.11.-)	13	93962	4	6.2	5.97
Gene.10133::comp2839	Putative tuberin	33	242948	3	2.4	6.03
Gene.7877::comp2202	Putative tpr	27	279132	5	2.8	4.81
Gene.2580::comp653	Putative myosin-10	24	230961	6	3.5	5.75
Gene.16843::comp4930	Meiotic checkpoint regulator cut4, putative	21	248934	7	5.4	6.62
Gene.17963::comp5299	Transcription-associated protein 1	17	232379	1	0.4	5.97

### Alignment of circulating *S*. *mekongi* protein and antigen sequences

**Fig 8:**
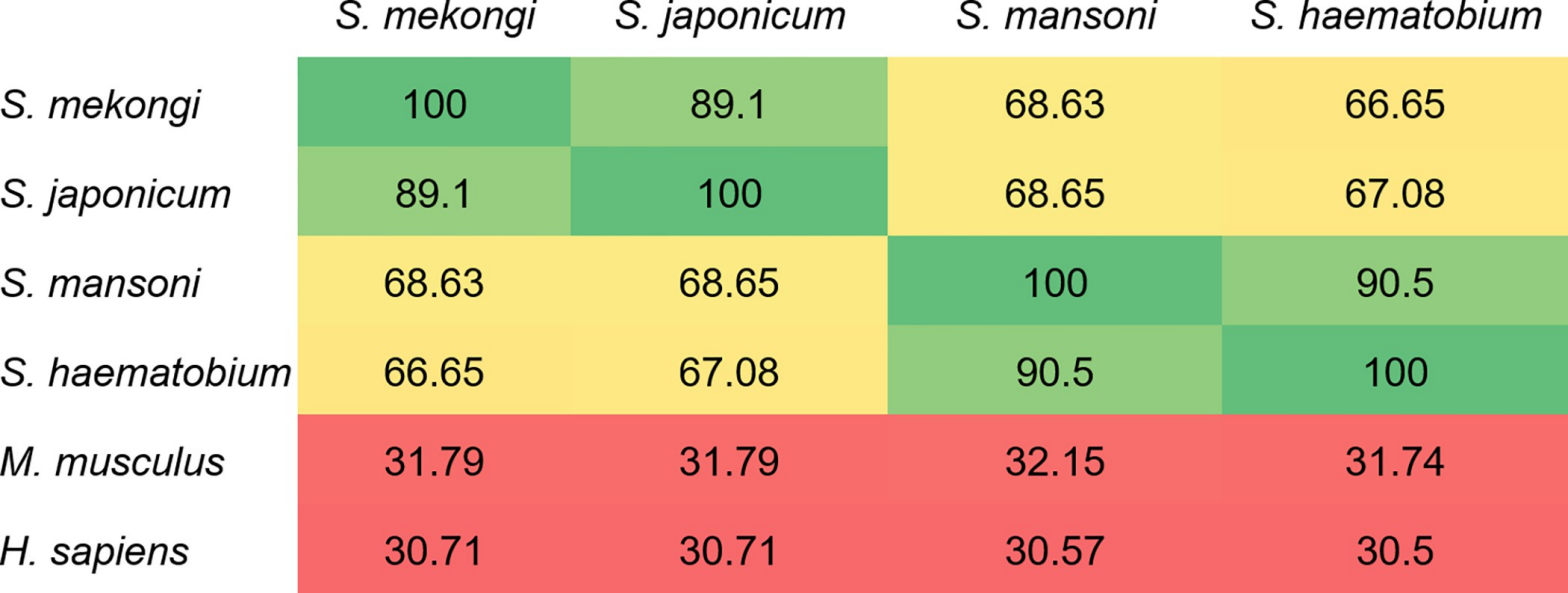
Percentage similarity in protein sequence alignment of putative tuberin among *Schistosoma* spp., *Mus musculus*, and *Homo sapiens*. All protein sequences were obtained from the non-redundant protein sequence databases of NCBI. Sequence alignment and identity calculations were performed using Clustal Omega software. Regions of highest similarity (green), high similarity (yellow), and lower similarity (red) are indicated.

Because the putative tuberin antigen was detected in mouse sera at all four infection timepoints, it may be a reliable biomarker for *S*. *mekongi* diagnosis throughout the course of infection, but especially the early stages. To evaluate the suitability of putative tuberin as a universal biomarker for schistosome infections, a comparison of *S*. *mekongi* putative tuberin protein sequence homology with homologs from the three most prevalent global *Schistosoma* spp., including *S*. *haematobium*, *S*. *mansoni*, and *S*. *japonicum*, was performed (Fig 8), and putative tuberin protein sequences from mice (*M*. *musculus*) and humans (*H*. *sapiens*) were also compared for homology with the schistosome proteins. In the homology analysis, putative tuberin of *S*. *mekongi* aligned with the highest similarity to a *S*. *japonicum* sequence (89.1%). Additionally, *S*. *mekongi* putative tuberin was similar to those of *S*. *mansoni* and *S*. *haematobium*, with 68.63% and 66.65% similarity, respectively. Because the putative tuberin sequence from *S*. *mekongi* had a low percentage similarity (less than 50%) with the murine and human proteins, it is not expected to show cross-reactivity during diagnosis and treatment. Hence, because putative tuberin is specific to all globally prevalent *Schistosoma* spp., it is a promising candidate biomarker for the reliable diagnosis of schistosomiasis and vaccine development.

### Discussion

Proteomic information for mouse sera before and after *S*. *mekongi* infection was explored in this study. After infection, differential mouse serum proteins were identified, including 19 up-regulated and 24 down-regulated proteins. At 14-days post-infection, immunoglobulin proteins were up-regulated, corresponding to a previous report that infection with *S*. *mansoni* stimulated transient immunoglobulin IgM responses in mice at 1-week post-infection [38]. In contrast, three serum proteins were down-regulated in infected mice: serine protease inhibitor (serpin) A3F, actin-alpha skeletal muscle, and 25-hydroxyvitamin D-1 alpha hydroxylase (1α-hydroxylase). Serpina3f (antichymotrypsin) plays a role in immune and inflammatory responses through the inhibition of chymotrypsin and cathepsin G [39, 40]. Because the serpina3 gene is regulated by various cytokines [41], its down-regulation may correspond to parasite infection and survival. Parasites regulate the host immune system by suppressing some immune-activated pathways to ensure their survival in the host [42]. Actin-alpha of skeletal muscle belongs to the actin family, which is important for the maintenance of cytoskeletons [43], and the down-regulation of actin-alpha is thought to be caused by changes to the skeletal muscles related to the pathophysiology of schistosomiasis [44]. The enzyme 1α-hydroxylase catalyzes the synthesis of active vitamin D. In a previous report, deficiency of vitamin D was typical in patients with hepatic fibrosis caused by schistosomiasis [45].

Immunoglobulin proteins were also up-regulated in mouse serum at 28-days post-infection. However, there were many down-regulated proteins, including alkaline phosphatase, macrophage erythroblast attacher, glutathione peroxidase 3, and complement C3. Alkaline phosphatase is an enzyme involved in skeletal mineralization [46], and it seems to play key roles in the anti-microbial activity of neutrophils by promoting the migration of neutrophils and the generation of ROS. Therefore, the down-regulation of alkaline phosphatase during *S*. *mekongi* infection may facilitate parasite survival [47]. However, this issue has to be explored more intensively prior to making a definite conclusion. Macrophage erythroblast attacher is an adhesion molecule involved in the formation of erythroblastic islands and the maintenance of hematopoietic stem cells [48, 49], whereas complement C3 is the most abundant complement system protein in serum, involved in inflammation, autoimmunity, and the host defense system [50]. Additionally, glutathione peroxidase was down-regulated in a manner corresponding to the decrease of glutathione peroxidase activity in *S*. *mansoni*-infected mice caused by schistosomiasis [51]. Both stromelysin-1 and complement factor B were up-regulated at 42-days post-infection. Stromelysin-1 belongs to matrix metalloproteinases, involved in bone growth and remodeling [52], and complement factor B relates to host inflammatory responses against parasitic invasion via the generation of proinflammatory molecules [53]. At 56-days post-infection, two proteins, inter-alpha-trypsin inhibitor heavy chain H3 and ceruloplasmin, were up-regulated: while inter-alpha-trypsin inhibitor heavy chain H3 is related to inflammation and carcinogenesis [54, 55], ceruloplasmin is a serum glycoprotein related to the acute phase of infection. The concentration of ceruloplasmin in serum was increased during the mediation of inflammation by cytokines [56, 57]; therefore, the up-regulation of these two proteins might be involved in mouse immune system attack against *S*. *mekongi*. In contrast, serum amyloid A4-protein and apolipoprotein M were down-regulated on day-56 post-infection. The down-regulation of serum amyloid might be caused by errors during switching between two distinct configurations related to the regulation of inflammation [58]. Apolipoprotein M (ApoM) is major component of high-density lipoprotein related to the modulation of immune responses and inflammation [59, 60]. The down-regulation of ApoM in the mice corresponds to a previous report in which the concentration of serum ApoM was described as decreased in patients with inflammation [61]. Although there is possibility that proteomic changes between uninfected (day 0) and infected mice (day 14, 28, 42 and 56 post-infection) might be cause by the age difference, using the same mice for both uninfected and infected conditions could reduce the biological variation in this study. In the proteomic analysis, Ig heavy chain V region MOPC 104E and serine protease inhibitors A3F were differential proteins found in infected mouse sera at all four timepoints; therefore, these proteins may be useful for studying host responses against *S*. *mekongi*.

The protein–protein interaction network reported five differential pathways in *S*. *mekongi*-infected mouse sera, including intermediate filament, vitamin D metabolism, complement activation, ubiquitin-dependent ER-associated degradation (ERAD), and serine-type endopeptidase inhibitor activity. Intermediate filaments are structural cytoskeletal components consisting of actin filaments and microtubules [62] and play roles in cell structural support as well as the regulation of many fundamental cellular processes [63]. The interactions between *S*. *mekongi*-infected mouse proteins and intermediate filaments may be related to the skeletal muscle changes seen with schistosomiasis as granuloma were detected in skeletal muscles of *S*. *mansoni* infected mice [44]. The active form of vitamin D is catalyzed by 1α-hydroxylase; therefore, the down-regulation of 1α-hydroxylase in *S*. *mekongi*-infected mouse sera may affect the vitamin D metabolism pathway [64]. In addition, complement proteins are associated with inflammation and the host defense system [50]. Ubiquitin is important for the tracking of target proteins via the ubiquitin-dependent ERAD pathway, a quality control process for the degradation of target misfolded ER proteins [65], and the ubiquitin-proteasome proteolytic pathway has been reported to play key roles during *S*. *mansoni* parasitic development [66, 67]. *S*. *mekongi* infection may affect serine-type endopeptidase inhibitor activity via the down-regulation of serpin, which is associated with the suppression of host immune responses during parasitic infection [42].

Parasitological and immunological diagnosis methods are not sensitive enough for the early detection of schistosomiasis because of infection features that include the absence of stool ova, low circulating antigen levels, and low specific antibody levels [6, 13]. Hence, circulating proteins and antigens of *S*. *mekongi* in infected mouse sera were identified in this study to obtain additional applicable biomarkers that can be applied to early schistosomiasis diagnosis. Fifty-four *S*. *mekongi* proteins were detected in infected mouse sera, one of which was an FMRFamide receptor detected at 14 days post-infection. FMRFamide-like peptides (FLPs) are key players in the neuromuscular biology of parasites; they are found throughout the flatworm nervous system, where they are involved in the excitation of muscle [68]. Many *S*. *mekongi* proteins were identified in infected mouse sera at 42-days post-infection. Voltage-dependent calcium (Ca^2+^) channel allows for Ca^2+^ influx, resulting in Ca^2+^-dependent responses in excitable tissue, such as muscles and nerves. In schistosomes, voltage-gated Ca^2+^ channels are sensitive to praziquantel, a drug commonly used in the treatment of schistosomiasis [69]. In helminths, several proteins are secreted using alternative export mechanism, membrane-bound vesicle, especially membrane proteins, cytoskeletal proteins, and heat shock proteins. However, the secretory mechanism is still unknown [70–72]. Niemann–Pick C1, a cholesterol-trafficking protein, belongs to the cholesterol-uptake pathway and is associated with Niemann–Pick disease type C in humans [73]. A homolog of Niemann–Pick Type C1 protein identified in *Plasmodium* parasites was reported to be important for the composition of parasite plasma membranes and the generation of digestive vacuoles [74]. Thus, Niemann–Pick Type C1 protein is considered to be involved in the formation of plasma membranes in *S*. *mekongi*. Schistosomes possess several components associated with the actin-based cytoskeletal system; e.g., dynein is a cytoskeletal motor consisting of light and heavy chains, the latter of which plays a key role in the movement of dynein [75, 76]. Hence, the up-regulated putative dynein heavy chain is expected to be involved in the cytoskeletal system of *S*. *mekongi*. NADH dehydrogenase (ubiquinone) Fe-S protein 2 is a NADH ubiquinone oxidoreductase subunit that functions in respiratory chains. Additionally, ubiquinone Fe-S protein 7 was identified in *S*. *mansoni* [77]. Neuropathy target esterase/swiss-cheese-related protein is an integral membrane protein in many species, including nematodes [78]. One of the proteins identified at 56-days post-infection, tensin, is an important binding component of the actin cytoskeleton [79]. The detection of aminopeptidase N is consistent with a previous study showing aminopeptidase activity in adult *S*. *rodhaini* [80]. Additionally, serine and threonine protein phosphatases play roles in the regulation of reproduction and growth in *S*. *japonicum* [81]. Rootletin, which was detected in this work, is expected to originate from miracidia in the ciliated larval stage [36]. Importantly, an enzyme identified at all four infection timepoints, kyphoscoliosis peptidase, plays key roles in the maturation and stabilization of neuromuscular junctions, resulting in normal muscle growth [82]. Kyphoscoliosis peptidase was detected in the cercariae and schistosomula of *S*. *japonicum* and is involved in the parasite’s invasion of hosts [83]. Kyphoscoliosis peptidase could not be observed in uninfected mice, thus it might be a promising specific biomarker for *S*. *mekongi* infection. The circulating protein data described in this work provide several candidate biomarkers for the diagnosis of *S*. *mekongi* infection, particularly kyphoscoliosis peptidase, voltage-dependent calcium (Ca^2+^) channel protein, and putative dynein heavy chain.

To provide information on immune complexes in the circulation, antigens of *S*. *mekongi* in infected mouse serum were identified. This information is useful for understanding parasitic immune evasion and for vaccine development. SDS-PAGE analysis revealed differential protein bands at 55 kDa and 26 kDa in the infected mouse serum, corresponding to the heavy-chain and light-chain of mouse immunoglobulin G (50 kDa and 25 kDa, respectively) [84]. Several circulating *S*. *mekongi* antigens were identified from infected mouse serum. Putative vasohibin, which is an angiogenesis inhibitor, was found to be up-regulated; thus *S*. *mekongi* may mediate angiogenesis inhibition in the host [85]. Tubulin tyrosine ligase-related protein is involved in the recruitment of microtubule-interacting proteins, and it was also identified in adult worms of *S*. *mansoni* [86, 87]. Moreover, suppressor of cytokine signaling 7 may be involved in the suppression of host immune responses via the inhibition of cytokine-inducible activator of transcription-mediated signal transduction [88, 89]. In schistosomes, protein kinase plays essential roles in growth, development, and host interaction; therefore, they have been considered as targets for drugs against parasitic diseases in many research efforts [90]. Serine/threonine protein phosphatases are important for the control of *S*. *japonicum* growth and reproduction [81], whereas cullin-3 is a core component of the E3 ubiquitin ligase complex and may play roles in the development of the reproductive organs of *S*. *mekongi* [24, 91]. The 5-hydroxytryptamine receptor 1 is generally a subtype of serotonin receptor [92]. In schistosomes, serotonin stimulates movement and motor activity [93]. In addition, *S*. *mansoni* responds to serotonin by activating serotonin receptors, with similar responses seen in the sporocyst stage and adult worms [94]. Putative tetratricopeptide repeat is a structural motif present inside the O-glycosyltransferase of schistosomes and is also highly expressed in female flukes [95]. Myosin X is involved in the movement of actin bundles, and the muscles of schistosomes contain both muscle-like myosin filaments and smooth muscle-like actin filaments [96].

Notably, putative tuberin was the only circulating antigen identified at all four infection timepoints. Tuberin, which is encoded by the expression of the tuberous sclerosis-2 gene [97], works together with hamartin to promote tumor suppression. In addition, a mutation of tuberin was found to lead to uncontrollable cell proliferation [98]. Therefore, tuberin may play important roles in the control of cell growth and proliferation in *S*. *mekongi*. To evaluate the specificity of a diagnosis based on tuberin, alignment and homology comparisons of tuberin protein sequences from *Schistosoma* spp. were made. While putative tuberin of *S*. *mekongi* had high similarity to tuberin sequences of other schistosomes, it was less similar to those of humans and mice. Therefore, the circulating antigen putative tuberin could be used as a specific target in schistosome immune-based diagnosis and vaccine development. Antigen-based detection is useful for diagnosis because it can discriminate active infections from past infections, and it can be used for evaluation during chemotherapy. However, the candidate biomarkers in this study were identified from mice sera; therefore, proteomic studies should be carefully performed in schistosomiasis patients. Additionally, the validation of synthetic potential biomarkers should be also performed in future. The identification of biomarkers in human and other host models could be beneficial for the early diagnosis of schistosomiasis.

## Conclusion

This study has provided proteomic information on differential proteins in *S*. *mekongi*-infected mouse sera. Circulating proteins and antigens of *S*. *mekongi* were discovered at different infection timepoints. Several additional proteins were identified as candidate biomarkers for the early diagnosis of schistosomiasis. The identification of these promising biomarkers is useful, and they may be applicable to the development of treatments, vaccines, and diagnostics for schistosomiasis.

## Supporting information

S1 Raw images The raw images of SDS-PAGE. (PDF)

S1 Table Identification of mouse serum protein. (XLSX)

S2 Table Identification of *S*. *mekongi* circulating protein. (XLSX)

S3 Table Identification of *S*. *mekongi* peptide sequences in infected mouse sera. (XLSX)

S4 Table Identification of *S*. *mekongi* circulating antigen. (XLSX)

S1 File Sequences of tuberin. (DOCX)

S1 Graphical abstract (TIF)

## References

[1] Gryseels B, Polman K, Clerinx J, Kestens L. Human schistosomiasis. Lancet. 2006;368(9541): 1106–1118. doi: 10.1016/S0140-6736(06)69440-3 16997665

[2] Steinmann P, Keiser J, Bos R, Tanner M, Utzinger J. Schistosomiasis and water resources development: systematic review, meta-analysis, and estimates of people at risk. Lancet Infect. Dis. 2006;6(7):411–425. doi: 10.1016/S1473-3099(06)70521-7 16790382

[3] Hotez PJ, Fenwick A, Savioli L, Molyneux DH. Rescuing the bottom billion through control of neglected tropical diseases. Lancet. 2009;373(9674):1570–1575. doi: 10.1016/S0140-6736(09)60233-6 19410718

[4] King CH. Parasites and poverty: the case of schistosomiasis. Acta Trop. 2010;113(2):95–104. doi: 10.1016/j.actatropica.2009.11.012 19962954 PMC2812649

[5] Kolářová L, Horák P, Skírnisson K, Marečková H, Doenhoff M. Cercarial dermatitis, a neglected allergic disease. Clin. Rev. Allergy Immunol. 2013;45(1):63–74. doi: 10.1007/s12016-012-8334-y 22915284

[6] McManus DP, Dunne DW, Sacko M, Utzinger J, Vennervald BJ, Zhou X. Schistosomiasis. Nat. Rev. Dis. Primers. 2018;4:13. doi: 10.1038/s41572-018-0013-8 30093684

[7] Voge M, Bruckner D, Bruce JI. *Schistosoma mekongi* sp. n. from man and animals, compared with four geographic strains of *Schistosoma japonicum*. J. Parasitol. 1978;577–584. doi: 10.2307/3279936 682060

[8] Muth S, Sayasone S, Odermatt-Biays S, Phompida S, Duong S, Odermatt P. *Schistosoma mekongi* in Cambodia and Lao People’s Democratic Republic. Adv. Parasitol. 2010;72:179–203. doi: 10.1016/S0065-308X(10)72007-8 20624532

[9] Attwood SW. Schistosomiasis in the Mekong region: epidemiology and phylogeography. Adv. Parasitol. 2001;50:87–152. doi: 10.1016/s0065-308x(01)50030-5 11757333

[10] Urbani C, Sinoun M, Socheat D, Pholsena K, Strandgaard H, Odermatt P, et al. Epidemiology and control of mekongi schistosomiasis. Acta Trop. 2002;82(2):157–168. doi: 10.1016/s0001-706x(02)00047-5 12020888

[11] McManus DP, Bergquist R, Cai P, Ranasinghe S, Tebeje BM, You H. Schistosomiasis-from immunopathology to vaccines. Semin Immunopathol. 2020;42:355–371. doi: 10.1007/s00281-020-00789-x 32076812 PMC7223304

[12] Weerakoon KG, Gobert GN, Cai P, McManus DP. Advances in the diagnosis of human schistosomiasis. Clin. Microbiol. Rev. 2015;28(4):939–967. doi: 10.1128/CMR.00137-14 26224883 PMC4548261

[13] Wang C, Chen L, Yin X, Hua W, Hou M, Ji M, et al. Application of DNA-based diagnostics in detection of schistosomal DNA in early infection and after drug treatment. Parasites Vectors. 2011;4(1):1–9. doi: 10.1186/1756-3305-4-164 21864384 PMC3177784

[14] Doenhoff MJ, Chiodini PL, Hamilton JV. Specific and sensitive diagnosis of schistosome infection: can it be done with antibodies. Trends Parasitol. 2004;20(1):35–39. doi: 10.1016/j.pt.2003.10.019 14700588

[15] Silva-Moraes V, Ferreira JMS, Coelho PMZ, Grenfell RFQ. Biomarkers for schistosomiasis: towards an integrative view of the search for an effective diagnosis. Acta Trop. 2014;132:75–79. doi: 10.1016/j.actatropica.2013.12.024 24412728

[16] Pearson MS, Tedla BA, Mekonnen GG, Proietti C, Becker L, Nakajima R, et al. Immunomics-guided discovery of serum and urine antibodies for diagnosing urogenital schistosomiasis: a biomarker identification study. Lancet Microbe. 2021;2(11):e617–e626. doi: 10.1016/S2666-5247(21)00150-6 34977830 PMC8683377

[17] Folayowon JO, Adebayo AS, Isokpehi RD, Anumudu CI. Bioinformatics evaluation of the homologues of *Schistosoma mansoni* biomarker proteins of bladder cancer in other *Schistosoma* species. bioRxiv. 2020. doi: 10.1101/2020.09.07.285767

[18] Kardoush MI, Ward BJ, Ndao M. Identification of candidate serum biomarkers for *Schistosoma mansoni* infected mice using multiple proteomic platforms. PLoS ONE. 2016;11(5):e0154465. doi: 10.1371/journal.pone.0154465 27138990 PMC4854390

[19] Zhang M, Fu Z, Li C, Han Y, Cao X, Han H, et al. Screening diagnostic candidates for schistosomiasis from tegument proteins of adult *Schistosoma japonicum* using an immunoproteomic approach. PLOS Negl. Trop. Dis. 2015;9(2):e0003454. doi: 10.1371/journal.pntd.0003454 25706299 PMC4338221

[20] Bi NN, Zhao S, Zhang JF, Cheng Y, Zuo CY, Yang GL, et al. Proteomics investigations of potential protein biomarkers in sera of rabbits infected with *Schistosoma japonicum*. Front. Cell. Infect. 2021;1270. doi: 10.3389/fcimb.2021.784279 35004354 PMC8729878

[21] Homsana A, Odermatt P, Southisavath P, Yajima A, Sayasone S. Cross-reaction of POC-CCA urine test for detection of *Schistosoma mekongi* in Lao PDR: a cross-sectional study. Infect. Dis. Poverty. 2020;9:114. doi: 10.1186/s40249-020-00733-z 32787912 PMC7424653

[22] Marti H, Halbeisen S, Bausch K, Nickel B, Neumayr A. Specificity of the POC-CCA urine test for diagnosing *S*. *mansoni* schistosomiasis. Travel Med. Infect. Dis. 2020;33:101473. doi: 10.1016/j.tmaid.2019.101473 31505266

[23] Thiangtrongjit T, Adisakwattana P, Limpanont Y, Dekumyoy P, Nuamtanong S, Chusongsang P, et al. Proteomic and immunomic analysis of *Schistosoma mekongi* egg proteins. Exp. Parasitol. 2018;191: 88–96. doi: 10.1016/j.exppara.2018.07.002 30009810

[24] Simanon N, Adisakwattana P, Thiangtrongjit T, Limpanont Y, Chusongsang P, Chusongsang Y, et al. Phosphoproteomics analysis of male and female *Schistosoma mekongi* adult worms. Sci. Rep. 2019;9(1):1–10. doi: 10.1038/s41598-019-46456-6 31292487 PMC6620315

[25] Reamtong O, Simanon N, Thiangtrongjit T, Limpanont Y, Chusongsang P, Chusongsang Y, et al. Proteomic analysis of adult *Schistosoma mekongi* somatic and excretory-secretory proteins. Acta Trop. 2020;202:105247. doi: 10.1016/j.actatropica.2019.105247 31672487

[26] Thiangtrongjit T, Simanon N, Adisakwattana P, Limpanont Y, Chusongsang P, Chusongsang Y, et al. Identification of low molecular weight proteins and peptides from *Schistosoma mekongi* worm, egg and infected mouse sera. Biomolecules. 2021;11(4):559. doi: 10.3390/biom11040559 33920436 PMC8070599

[27] Alharbi RA. Proteomics approach and techniques in identification of reliable biomarkers for diseases. Saudi J. Biol. Sci. 2020;27(3):968–974. doi: 10.1016/j.sjbs.2020.01.020 32127776 PMC7042613

[28] Al-Amrani S, Al-Jabri Z, Al-Zaabi A, Alshekaili J, Al-Khabori M. Proteomics: concepts and applications in human medicine. World J. Biol. Chem. 2021;12(5):57. doi: 10.4331/wjbc.v12.i5.57 34630910 PMC8473418

[29] Galassie AC, Link AJ. Proteomic contributions to our understanding of vaccine and immune responses. Proteomics Clin. Appl. 2015;9(11–12):972–989. doi: 10.1002/prca.201500054 26172619 PMC4713355

[30] Amiri-Dashatan N, Koushki M, Abbaszadeh HA, Rostami-Nejad M, Rezaei-Tavirani M. Proteomics applications in health: biomarker and drug discovery and food industry. Iran J. Pharm. Res. 2018;17(4):1523. PMC6269565 30568709

[31] Chienwichai P, Tipthara P, Tarning J, Limpanont Y, Chusongsang P, Chusongsang Y, et al. Metabolomics reveal alterations in arachidonic acid metabolism in *Schistosoma mekongi* after exposure to praziquantel. PLOS Negl. Trop. Dis. 2021;15(9):e0009706. doi: 10.1371/journal.pntd.0009706 34473691 PMC8412319

[32] Fish-Low CY, Than LTL, Ling KH, Lin Q, Sekawi Z. Plasma proteome profiling reveals differentially expressed lipopolysaccharide-binding protein among leptospirosis patients. J. Microbiol. Immunol. Infect. 2020;53(1):157–162. doi: 10.1016/j.jmii.2018.12.015 31029530

[33] Ma G, Wang P, Yang Y, Wang W, Ma J, Zhou L, et al. emPAI‐assisted strategy enhances screening and assessment of *Mycobacterium tuberculosis* infection serological markers. Microb. Biotechnol. 2021;14(4):1827–1838. doi: 10.1111/1751-7915.13829 34173722 PMC8313264

[34] Almagro Armenteros JJ, Tsirigos KD, Sønderby CK, Petersen TN, Winther O, Brunak S, et al. SignalP 5.0 improves signal peptide predictions using deep neural networks. Nat. Biotechnol. 2019;37:420–423. doi: 10.1038/s41587-019-0036-z 30778233

[35] Bendtsen JD, Kiemer L, Fausbøll A, Brunak S. Non-classical protein secretion in bacteria. BMC Microbiol. 2005;5:58. doi: 10.1186/1471-2180-5-58 16212653 PMC1266369

[36] Wang T, Zhao M, Rotgans BA, Strong A, Liang D, Ni G, et al. Proteomic analysis of the *Schistosoma mansoni* miracidium. PLoS ONE. 2016;11(1):e0147247. doi: 10.1371/journal.pone.0147247 26799066 PMC4723143

[37] Kifle DW, Pearson MS, Becker L, Pickering D, Loukas A, Sotillo J. Proteomic analysis of two populations of *Schistosoma mansoni*-derived extracellular vesicles: 15k pellet and 120k pellet vesicles. Mol. Biochem. Parasitol. 2020;236:111264. 32014446 10.1016/j.molbiopara.2020.111264

[38] Toy L, Pettit M, Wang YF, Hedstrom R, McKerrow JH. The immune response to stage-specific proteolytic enzymes of *Schistosoma mansoni*. In molecular paradigms for eradicating helminthic parasites. Proceedings of an Upjohn-UCLA symposium, steamboat springs, Colorado, USA, 24–31 January 1987. 1987; 85–103.

[39] Heit C, Jackson BC, McAndrews M, Wright MW, Thompson DC, Silverman GA, et al. Update of the human and mouse SERPINgene superfamily. Hum. Genomics. 2013;7(1):1–14. doi: 10.1186/1479-7364-7-22 24172014 PMC3880077

[40] Law RH, Zhang Q, McGowan S, Buckle AM, Silverman GA, Wong W, et al. An overview of the serpin superfamily. Genome Biol. 2006;7(5):1–11. doi: 10.1186/gb-2006-7-5-216 16737556 PMC1779521

[41] Sánchez-Navarro A, González-Soria I, Caldiño-Bohn R, Bobadilla NA. An integrative view of serpins in health and disease: The contribution of SerpinA3. Am. J. Physiol., Cell Physiol. 2021;320(1):106–118. doi: 10.1152/ajpcell.00366.2020 33112643

[42] Maizels RM, McSorley HJ. Regulation of the host immune system by helminth parasites. J. Allergy Clin. Immunol. 2016;138(3):666–675. doi: 10.1016/j.jaci.2016.07.007 27476889 PMC5010150

[43] Laing NG, Dye DE, Wallgren-Pettersson C, Richard G, Monnier N, Lillis S, et al. Mutations and polymorphisms of the skeletal muscle α-actin gene (ACTA1). Hum. Mutat. 2009;30(9):1267–1277. doi: 10.1002/humu.21059 19562689 PMC2784950

[44] Fidelis TAA, Brasileiro-Filho G, Parreiras PM, Coelho PMZ, Araujo N, Chaud MV, et al. *Schistosoma mansoni* granulomas in the skeletal striated muscles in the murine model of neuroschistosomiasis: histological findings. Mem. Inst. Oswaldo Cruz. 2020;115. doi: 10.1590/0074-02760190383 32401896 PMC7212994

[45] Zhou LY, Wu YM, Zhang LF. Serum vitamin D expression in advanced schistosomiasis patients with hepatic fibrosis and its association with disease progression. Chin. J. Schistosomiasis Control. 2019;32(3):304–307. doi: 10.16250/j.32.1374.2019122 32468796

[46] Millan JL. Alkaline phosphatases structure, substrate specificity and functional relatedness to other members of a large superfamily of enzymes. Purinergic Signal. 2006;2(2):335–341. doi: 10.1007/s11302-005-5435-6 18404473 PMC2254479

[47] Li H, Zhao Y, Li W, Yang J, Wu H. Critical role of neutrophil alkaline phosphatase in the antimicrobial function of neutrophils. Life Sci. 2016;157:152–157. doi: 10.1016/j.lfs.2016.06.005 27287680

[48] Hanspal M, Hanspal JS. The association of erythroblasts with macrophages promotes erythroid proliferation and maturation: a 30-kD heparin-binding protein is involved in this contact. Blood. 1994;84(10):3494–3504. doi: 10.1182/blood.V84.10.3494.3494 7949103

[49] Wei Q, Pinho S, Dong S, Pierce H, Li H, Nakahara F, et al. MAEA is an E3 ubiquitin ligase promoting autophagy and maintenance of haematopoietic stem cells. Nat. Commun. 2021;12(1):1–13. doi: 10.1038/s41467-021-22749-1 33947846 PMC8097058

[50] Copenhaver M, Yu CY, Hoffman RP. Complement components, C3 and C4, and the metabolic syndrome. Curr. Diabetes Rev. 2019;15(1):44–48. doi: 10.2174/1573399814666180417122030 29663892

[51] Gharib B, Abdallahi OMS, Dessein H, De Reggi M. Development of eosinophil peroxidase activity and concomitant alteration of the antioxidant defenses in the liver of mice infected with *Schistosoma mansoni*. J. Hepatol. 1999;30(4):594–602. doi: 10.1016/S0168-8278(99)80189-5 10207800

[52] Lee M, Shimizu E, Krane SM, Partridge NC. Bone proteinases. In: Bilezikian JP, Raisz LG, Martin TJ, editors. Principles of Bone Biology; 2008. pp. 367–384.

[53] Shao S, Sun X, Chen Y, Zhan B, Zhu X. Complement evasion: an effective strategy that parasites utilize to survive in the host. Front. Microbiol. 2019;10:532. doi: 10.3389/fmicb.2019.00532 30949145 PMC6435963

[54] Hamm A, Veeck J, Bektas N, Wild PJ, Hartmann A, Heindrichs U, et al. Frequent expression loss of Inter-alpha-trypsin inhibitor heavy chain (ITIH) genes in multiple human solid tumors: a systematic expression analysis. BMC cancer. 2008;8(1):1–15. doi: 10.1186/1471-2407-8-25 18226209 PMC2268946

[55] Jiang X, Bai XY, Li B, Li Y, Xia K, Wang M, et al. Plasma inter-alpha-trypsin inhibitor heavy chains H3 and H4 serve as novel diagnostic biomarkers in human colorectal cancer. Disease markers. 2019;5069614. doi: 10.1155/2019/5069614 31481982 PMC6701429

[56] Gitlin JD. Transcriptional regulation of ceruloplasmin gene expression during inflammation. J. Biol. Chem. 1988;263(13):6281–6287. doi: 10.1016/S0021-9258(18)68783-6 3360784

[57] Mohiuddin SS, Manjrekar P. Role of ceruloplasmin as a low grade chronic inflammatory marker and activated innate immune system in pathogenesis of diabetes mellitus. J. Diabetes Metab. Disord. Control. 2018;5:148–153. doi: 10.15406/jdmdc.2018.05.00155

[58] Wang W, Khatua P, Hansmann UH. Cleavage, downregulation, and aggregation of serum amyloid A. J. Phys. Chem. B. 2020;124(6):1009–1019. doi: 10.1021/acs.jpcb.9b10843 31955564 PMC7346682

[59] Wang M, Luo GH, Liu H, Zhang YP, Wang B, Di DM, et al. Apolipoprotein M induces inhibition of inflammatory responses via the S1PR1 and DHCR24 pathways. Mol. Med. Rep. 2019;19(2):1272–1283. doi: 10.3892/mmr.2018.9747 30569161

[60] Georgila K, Vyrla D, Drakos E. Apolipoprotein A-I (ApoA-I), immunity, inflammation and cancer. Cancers. 2019;11(8):1097. doi: 10.3390/cancers11081097 31374929 PMC6721368

[61] Kumaraswamy SB, Linder A, Åkesson P, Dahlbäck B. Decreased plasma concentrations of apolipoprotein M in sepsis and systemic inflammatory response syndromes. Crit. Care. 2012;16(2):1–7. doi: 10.1186/cc11305 22512779 PMC3681389

[62] Herrmann H, Aebi U. Intermediate filaments: structure and assembly. Cold Spring Harb. Perspect. Biol. 2016;8(11):a018242. doi: 10.1101/cshperspect.a018242 27803112 PMC5088526

[63] Bernot KM, Coulombe PA. Intermediate filaments. In: Lennarz WJ, Lane MD, editors. Encyclopedia of Biological Chemistry; 2013. pp. 631–636.

[64] Hewison M, Zehnder D, Bland R, Stewart PM. 1alpha-Hydroxylase and the action of vitamin D. J. Mol. Endocrinol. 2000;25(2):141–8. doi: 10.1677/jme.0.0250141 11013342

[65] Qi L, Tsai B, Arvan P. New insights into the physiological role of endoplasmic reticulum-associated degradation. Trends Cell Biol. 2017;27(6):430–440. doi: 10.1016/j.tcb.2016.12.002 28131647 PMC5440201

[66] Guerra-Sá R, Castro-Borges W, Evangelista EA, Kettelhut IC, Rodrigues V. *Schistosoma mansoni*: functional proteasomes are required for development in the vertebrate host. Exp. Parasitol. 2005;109(4):228–236. doi: 10.1016/j.exppara.2005.01.002 15755420

[67] Castro-Borges W, Cartwright J, Ashton PD, Braschi S, Guerra Sa R, Rodrigues V, et al. The 20S proteasome of *Schistosoma mansoni*: a proteomic analysis. Proteomics. 2007;7(7):1065–1075. doi: 10.1002/pmic.200600166 17390295

[68] Novozhilova E, Kimber MJ, Qian H, McVeigh P, Robertson AP, Zamanian M, et al. FMR-Famide-like peptides (FLPs) enhance voltage-gated calcium currents to elicit muscle contraction in the human parasite *Schistosoma mansoni*. PLoS Negl. Trop. Dis. 2010;4(8):e790. doi: 10.1371/journal.pntd.0000790 20706630 PMC2919380

[69] Jeziorski MC, Greenberg RM. Voltage-gated calcium channel subunits from platyhelminths: potential role in praziquantel action. Int. J. Parasitol. 2006;36(6):625–632. doi: 10.1016/j.ijpara.2006.02.002 16545816 PMC3788357

[70] Colombo M, Raposo G, Théry C. Biogenesis, secretion, and intercellular interactions of exosomes and other extracellular vesicles. Annu. Rev. Cell Dev. Biol. 2014;30:255–289. doi: 10.1146/annurev-cellbio-101512-122326 25288114

[71] Samoil V, Dagenais M, Ganapathy V, Aldridge J, Glebov A, Jardim A. Vesicle-based secretion in schistosomes: analysis of protein and microRNA (miRNA) content of exosome-like vesicles derived from *Schistosoma mansoni*. Scientific reports. 2018;8(1):1–16. doi: 10.1038/s41598-018-21587-4 29459722 PMC5818524

[72] Théry C. Exosomes: secreted vesicles and intercellular communications. F1000 biology reports. 2011;3. doi: 10.3410/B3-15 21876726 PMC3155154

[73] Li X, Wang J, Coutavas E, Shi H, Hao Q, Blobel G. Structure of human Niemann-Pick C1 protein. Proc. Natl. Acad. Sci. 2016;113(29):8212–8217. doi: 10.1073/pnas.1607795113 27307437 PMC4961162

[74] Istvan ES, Das S, Bhatnagar S, Beck JR, Owen E, Llinas M, et al. Plasmodium Niemann-Pick type C1-related protein is a druggable target required for parasite membrane homeostasis. Elife. 2019;8:e40529. doi: 10.7554/eLife.40529 30888318 PMC6424564

[75] Asai DJ, Koonce MP. The dynein heavy chain: structure, mechanics and evolution. Trends Cell Biol. 2001;11(5):196–202. doi: 10.1016/s0962-8924(01)01970-5 11316608

[76] Jones MK, Gobert GN, Zhang L, Sunderland P, McManus DP. The cytoskeleton and motor proteins of human schistosomes and their roles in surface maintenance and host-parasite interactions. Bioessays. 2004;26(7):752–765. doi: 10.1002/bies.20058 15221857

[77] Abreu FC, Mota EA, Pereira RV, Oliveira VF, Costa MP, Gomes MDS, et al. Differential expression profiles of miRNAs and their putative targets in *Schistosoma mansoni* during its life cycle. Mem. Inst. Oswaldo Cruz. 2021;116. doi: 10.1590/0074-02760200326 34008737 PMC8128373

[78] Lush MJ, Li Y, Read DJ, Willis AC, Glynn P. Neuropathy target esterase and a homologous Drosophila neuro-degeneration-associated mutant protein contain a novel domain conserved from bacteria to man. Biochem. J. 1998;332(1):1–4. doi: 10.1042/bj3320001 9576844 PMC1219444

[79] Lo SH. Tensin. Int. J. Biochem. Cell Biol. 2004;36(1):31–34. doi: 10.1016/s1357-2725(03)00171-7 14592531

[80] Fripp PJ. The histochemical localization of leucine aminopeptidase in *Schistosoma rodhaini*. Comp. Biochem. Physio. 1967;20(1):307–309. doi: 10.1016/0010-406X(67)90744-X

[81] Zhao L, Lu Z, He X, Mughal MN, Fang R, Zhou Y, et al. Serine/threonine protein phosphatase 1 (PP1) controls growth and reproduction in *Schistosoma japonicum*. FASEB J. 2018;32(12):6626–6642. doi: 10.1096/fj.201800725R 29879373

[82] Blanco G, Coulton GR, Biggin A, Grainge C, Moss J, Barrett M, et al. The kyphoscoliosis (ky) mouse is deficient in hyper-trophic responses and is caused by a mutation in a novel muscle-specific protein. Hum. Mol. Genet. 2001;10(1):9–16. doi: 10.1093/hmg/10.1.9 11136708

[83] Liu M, Ju C, Du XF, Shen HM, Wang JP, Li J, et al. Proteomic analysis on cercariae and schistosomula in reference to potential proteases involved in host invasion of *Schistosoma japonicum* larvae. J. Proteome Res. 2015;14(11):4623–4634. doi: 10.1021/acs.jproteome.5b00465 26370134

[84] Chiu ML, Goulet DR, Teplyakov A, Gilliland GL. Antibody structure and function: the basis for engineering therapeutics. Antibodies. 2019;8(4):55. doi: 10.3390/antib8040055 31816964 PMC6963682

[85] Venancio TM, DeMarco R, Almeida GT, Oliveira KC, Setubal JC, Verjovski-Almeida S. Analysis of *Schistosoma mansoni* genes shared with Deuterostomia and with possible roles in host interactions. BMC Genom. 2007;8(1):1–15. doi: 10.1186/1471-2164-8-407 17996068 PMC2194728

[86] Szyk A, Deaconescu AM, Piszczek G, Roll-Mecak A. Tubulin tyrosine ligase structure reveals adaptation of an ancient fold to bind and modify tubulin. Nat. Struct. Mol. Biol. 2011;18(11):1250–1258. doi: 10.1038/nsmb.2148 22020298 PMC3342691

[87] Nawaratna SS, McManus DP, Moertel L, Gobert GN, Jones MK. Gene atlasing of digestive and reproductive tissues in *Schistosoma mansoni*. PLoS Negl. Trop. Dis. 2011;5(4):e1043. doi: 10.1371/journal.pntd.0001043 21541360 PMC3082511

[88] Martens N, Uzan G, Wery M, Hooghe R, Hooghe-Peters EL, Gertler A. Suppressor of cytokine signaling 7 inhibits prolactin, growth hormone, and leptin signaling by interacting with STAT5 or STAT3 and attenuating their nuclear translocation. J. Biol. Chem. 2005;280(14):13817–13823. doi: 10.1074/jbc.M411596200 15677474

[89] Ouaissi A. Regulatory cells and immunosuppressive cytokines: parasite-derived factors induce immune polarization. J. Biomed. Biotechnol. 2007;4:89–114. doi: 10.1155/2007/94971 17597838 PMC1893014

[90] Wu K, Zhai X, Huang S, Jiang L, Yu Z, Huang J. Protein kinases: potential drug targets against *Schistosoma japonicum*. Front. Cell. Infect. 2021;11. doi: 10.3389/fcimb.2021.691757 34277472 PMC8282181

[91] Dubiel W, Dubiel D, Wolf DA, Naumann M. Cullin 3-based ubiquitin ligases as master regulators of mammalian cell differentiation. Trends Biochem. Sci. 2018;43(2):95–107. doi: 10.1016/j.tibs.2017.11.010 29249570 PMC5801050

[92] Pithadia AB, Jain SM. 5-Hydroxytryptamine receptor subtypes and their modulators with therapeutic potentials. J. Clin. Med. 2009;1(2):72. doi: 10.4021/jocmr2009.05.1237 22505971 PMC3318857

[93] Patocka N, Sharma N, Rashid M, Ribeiro P. Serotonin signaling in *Schistosoma mansoni*: A Serotonin–activated G protein-coupled receptor controls parasite movement. PLOS Pathog. 2014;10(1):e1003878. doi: 10.1371/journal.ppat.1003878 24453972 PMC3894222

[94] Boyle JP, Zaide JV, Yoshino TP. *Schistosoma mansoni*: effects of serotonin and serotonin receptor antagonists on motility and length of primary sporocysts in vitro. Exp. Parasitol. 2000;94(4):217–226. doi: 10.1006/expr.2000.4500 10831389

[95] Phuphisut O, Ajawatanawong P, Limpanont Y, Reamtong O, Nuamtanong S, Ampawong S, et al. Transcriptomic analysis of male and female *Schistosoma mekongi* adult worms. Parasites Vectors. 2018;11(1):1–16. doi: 10.1186/s13071-018-3086-z 30201055 PMC6131826

[96] Sulbarán G, Alamo L, Pinto A, Márquez G, Méndez F, Padrón R, et al. An invertebrate smooth muscle with striated muscle myosin filaments. Proc. Natl. Acad. Sci. 2015;112(42):5660–5668. doi: 10.1073/pnas.1513439112 26443857 PMC4620896

[97] Wienecke R, König A, DeClue JE. Identification of tuberin, the tuberous sclerosis-2 product. Tuberin possesses specific Rap1GAP activity. J. Biol. Chem. 1995;270(27):16409–16414. doi: 10.1074/jbc.270.27.16409 7608212

[98] Jozwiak J. Hamartin and tuberin: working together for tumour suppression. Int. J. Cancer. 2006;118(1):1–5. doi: 10.1002/ijc.21542 16206276

