# Supplementary material for: Discovery of Schistosoma mekongi circulating proteins and antigens in infected mouse sera
Source: PLoS One. 2022 Oct 13;17(10):e0275992. doi: 10.1371/journal.pone.0275992 (PMC9562170; doi:10.1371/journal.pone.0275992)
Supplement: S1 Raw images — (PDF) [file pone.0275992.s001.pdf]

Fig.1. SDS-PAGE analysis of *S. mekongi*-infected mouse serum at 0- (pre-infection), 14-, 28-, 42-, and 56-days post-infection. M: marker; No. 1-3: mice no. 1-3. The 12 horizontal sections represent excised protein bands used in mass spectrometric analysis.

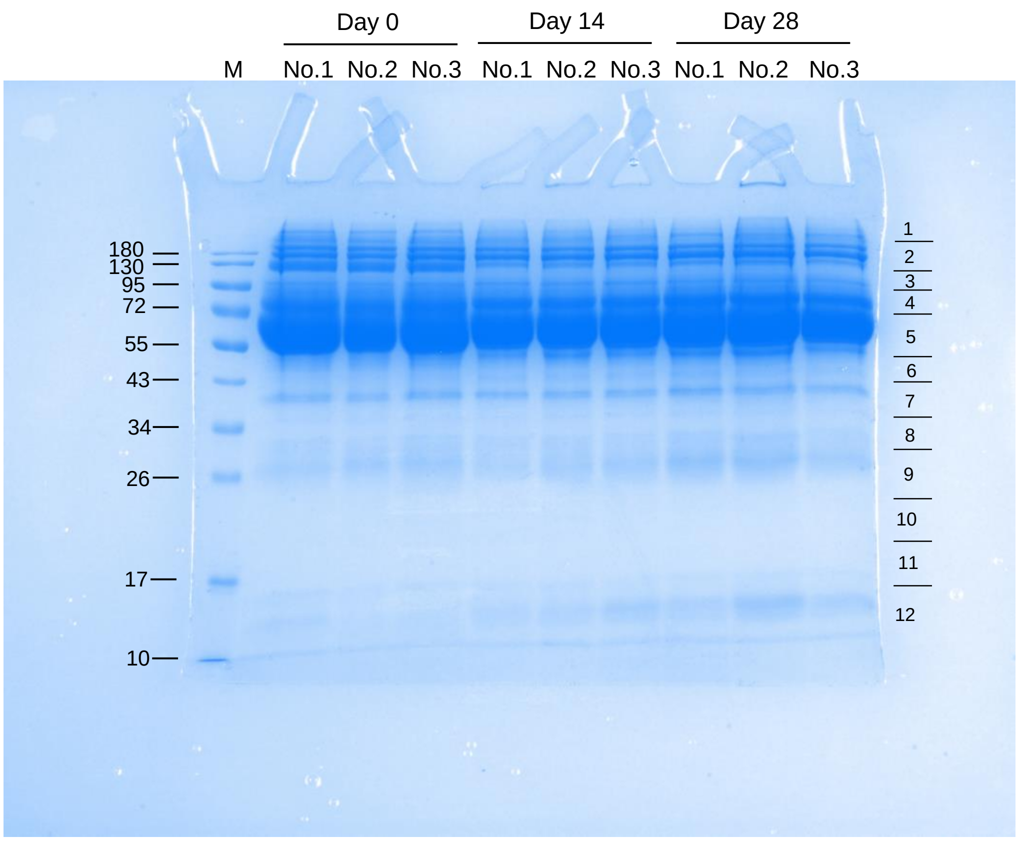

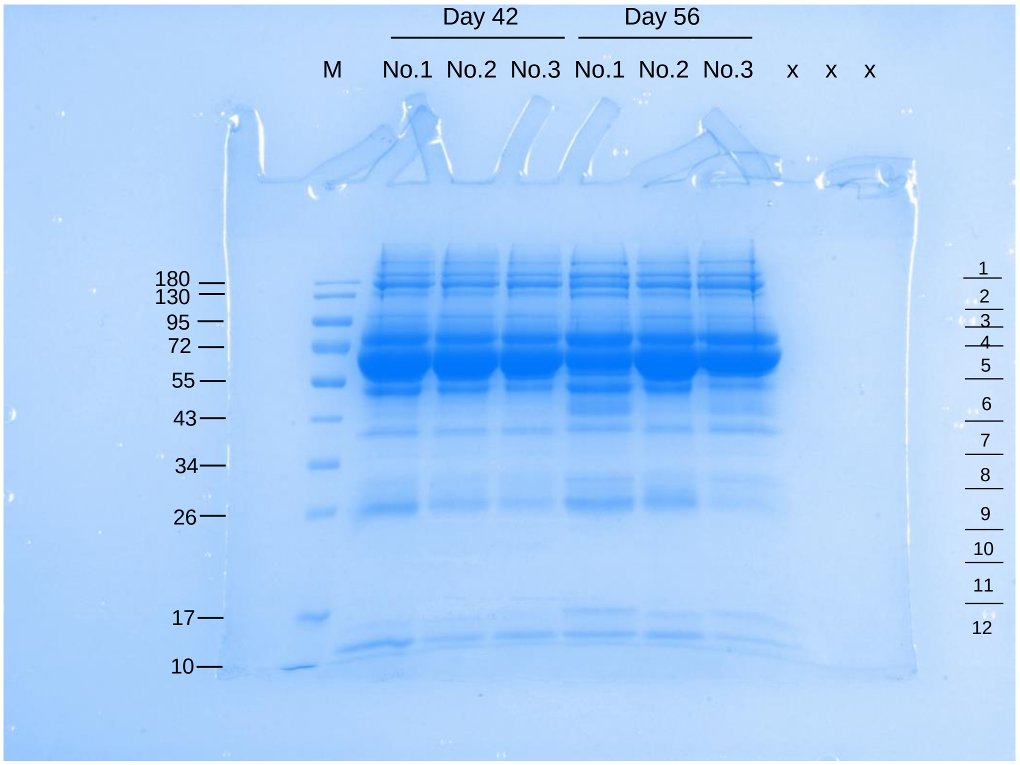

Method used to capture the image: Molecular Image® Gel Doc™ XR+ with image Lab™ Software

Fig 6. SDS-PAGE analysis of circulating antigens from immune complexes in sera from mice with and without S. mekongi infection. Immune complexes were enriched using protein A/G magnetic beads and separated with 12% SDS-PAGE. M: Marker; NI: Uninfected; 14-, 28-, 42-, and 56-days post-infection. The 10 horizontal sections represent regions excised for mass spectrometric analysis.

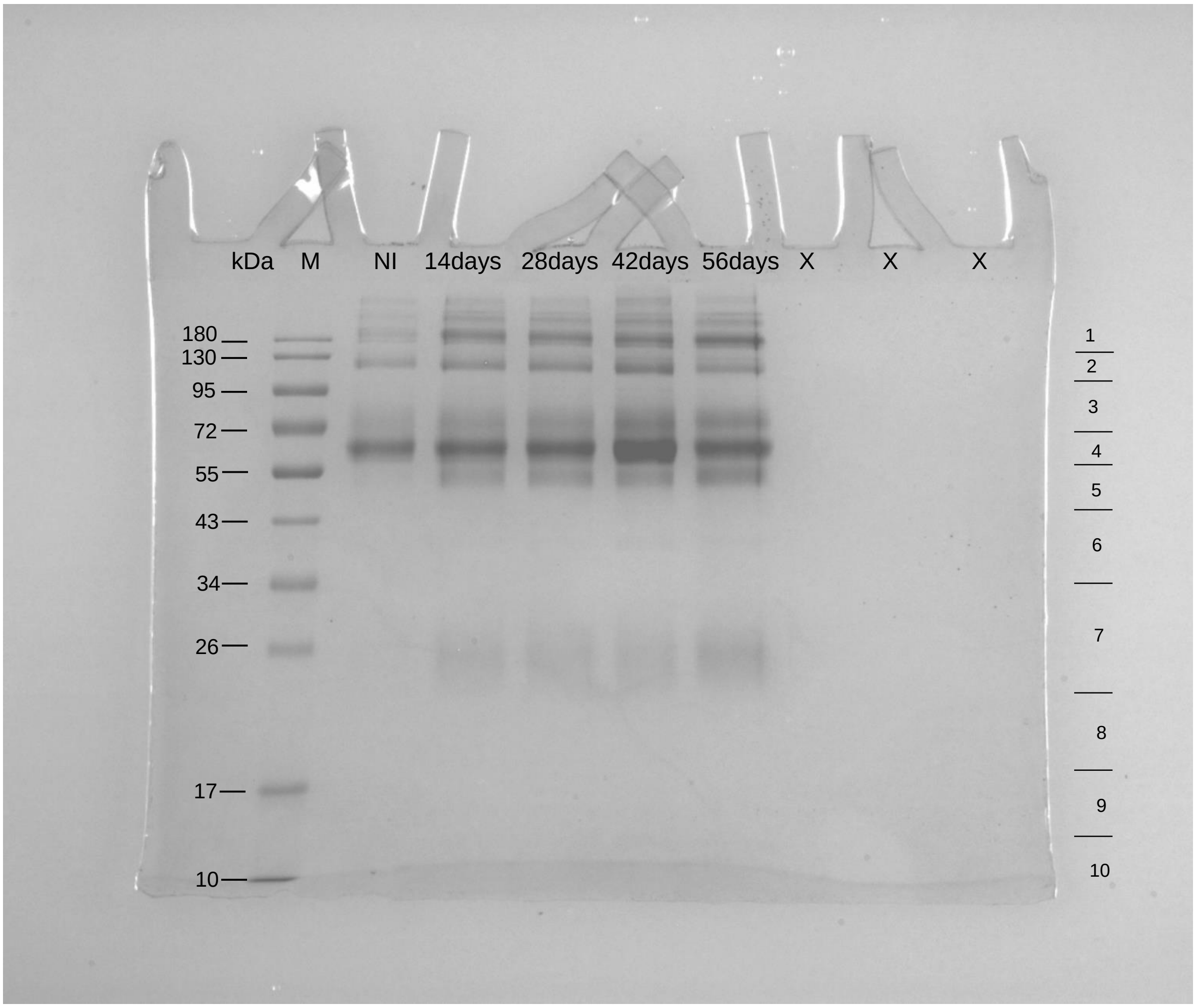

Method used to capture the image: Molecular Image® Gel Doc™ XR+ with image Lab™ Software
