# Supplementary material for: Discovery of Schistosoma mekongi circulating proteins and antigens in infected mouse sera
Source: PLoS One. 2022 Oct 13;17(10):e0275992. doi: 10.1371/journal.pone.0275992 (PMC9562170; doi:10.1371/journal.pone.0275992)
Supplement: S1 File — (DOCX) [file pone.0275992.s006.docx]

**Schistosoma mekongi tuberin**

MGEDESIGDVSLVLLQALLRFLVSESFKVEWSQLQYEQHLMQLWFLFEKLKHSYLPVIFP

CLSPSYSIYSPSSETNNTFQLTSIILHDYPIHPSRLSYYQLELIYWLASFVYTGPRSSSL

TVSSDGGPSSNSVTHGSVVGTTGASFNLSGSFPWDTHGDPNDFLLSRHFRSFIGCPGHGD

FESGSYSKANSYSKLSTSPVQTSGYIRYGDDNFSPMSFQELSDLASRNISPKPDNYFSKN

NSLIPSAFQTSPPLQKFIFTEPILNYSEKVVSMVHEVLYGCRQNINLVQDILHQALLLPL

HCHKAIFFVIIVYGSWLENKHNRPIFMQALDTPMKNSDQLLRSINSSQSTEENRALQINN

HLSDLKQIQQSQVGNSPTNKTKPEDNADDPEELKGCLQNTIQIMLENMANVFYASSLDVS

FSGPQNTNRVATNTVDYTKHQIDLCRLVLQVFQFASNSNELTCETWSKLLSILMDIMSRT

MINTSPTNVQNYKWLLNNKLTQNLFQTLNGALLRASLFSTISSEPWDQCLNVYSKLMHWP

SLIIEWKKVMRMLTCTMAKLVYGVDLSDLPQEKKRPRFKRLAASTNSANRARPKSLTESA

LNYSNNNNNNNPDINSSTSVLLSASVPYPSALSIVNFHQNSLDDNLMLNSGYTEPDNNIS

DNRDISRDHSKNMFATCSDVKALQNHNHRPSIEKWTSETVQHETTITNEMNNMINNDNAR

SHTSINYDGVADDESNLEEFSDDSTVMNNIKHKHNSQSIPQDLLTKTELVQLESGAKDKT

LISERSRLSFVRRATSEIALEVKFGLDSPESPVRTYFSESQGVNNLDIPRSYDRLNNGVP

NLQLCRQDTLIATQEVSVGNGSSKVGLAAENDQLNTVDVPRCILAGGPAFGWTNESVIAC

WRRFLGVLGNFHKITNPTTLSDIFSYLIELTTCLLKIREYQTVPCSTDTCIQPIPHFLPP

IDFISPILFDSMNLSEDFLEAKQIAIRALIDIAVCFNDGCPNLELIARFFHLIHRISVTK

HEKYIFEVVRSCNMRMFVSPLPSTHLLILDFLSNVKVVLANSNPGTEIPRSDALSIVLSL

LCYPYHFNRLESIDPMSMELKTIACQGLTSHLVHILTHAVLSDPSAEVRCLAITGLSIFC

VTELMNSSSKESGSVSSTGMNSLFFDAIIILLGMMRFQNRIVSIVAVDMINMLADYCHLL

SLCDIKLSCLVLLSLAWTLYSAWDNANLNTISSTDKQFFLSLFQAIIEWSMHIPYDQLIM

KFDNKTGSSLPALNLLDTVIEVLCCVMSNDTNSQSSINSQNSNITQPKYLPSIYFTDPLL

EHIGTCKQSIDITLFYASESELDLSTLTAKYWIGTNFENTSIESMYHNEMNHATESVRLA

ARMTISHLLNHIDQFPVSKHGAQLNTSIQEHHDQSAYSISTQKHYCANTVDDVSELTADI

LERNNVQIFVLNRSVILTFISLPIAVKSPTQLKDTSDSTADDKFDAALSNELNVELYNAA

YSYLPLSYLNSNSMEKISTKSGLVTDKFYTRIITRDLSGKYCWDASYLYGSIYDYMERRK

DNQLQSDELTNTNVSLMVDSNDLDLHLSRDPPPCPPPRVNPPSQPPLTNENTSHIVDTFD

RLNDVLRDLALTSPECSISWCTLKPSEYENVKNLSDKEATARLNMEKMTYDQISAQCQMD

DDTVHRKLADVDVLSLYDSHSLQPTGHKYSLRHTIRSMINNRNPNEFESIKQNTSQFYNS

RHLLNQLGYVSWRYRPHVDLLQKSPALVRELKHLDNLGSRETHKFAVFYVGAGQEDKQSI

LANQTTSLEFENFVAGLGWEIDLLTHKGFRGGLERSGRAGLSTPYYVTSTLEVIYHVSTR

MPSSTHEDLKYKHLGNDEVMIIWNENSRAFRRSVLRTQFGDVLIIISPLLNGLYKVEVRR

EAQIGLFGPIVENAVLCANVLPGLVRATAINASRAVQAIKPGYRRPYEDRASSLQQIVSK

HTLSTSFEEYTESILFPSSKTVYYDPSSGMNKATGVKFSLVPHNKQESLHNYASTSTSSP

VPNNVNSNNDHNALKHNHVTLTFSTRQKEEITSVALCQPTSAHLRGSSVSRSRGKLPGYQ

YVNNLNSNPISAVSQSPLKTNLPNVHPTENSDNYRMSNPVTSPRLFRGLKARRHSGSSKI

N

**Ral GTPase-activating protein subunit alpha-1 [Schistosoma japonicum]**

**GenBank: KAH8861019.1**

>KAH8861019.1 Ral GTPase-activating protein subunit alpha-1 [Schistosoma japonicum]

MFRRHRPSDISTLKSRKKFLSDKESTHKIKHLKILIDNLPHDELQPFFVENSSLIFQEYQDREKCNEQPK

RSHVQPGRLSVDARGQVIEESRISNVVAPRERVALLTAAAIEEDESTDDVSLVLFQALLRFLVSESFKVE

WSQLRYEQHLMQLWFLFEKLKYSYLPVIFPCLSPLYSIYSPLSEVNNTFQLTSNILHDYPIHPSRLSYYQ

LELVYWLASFVYTGPRSSSHTVSSDGGPSSNSVTHGSVVGTTGTSFNVNGSFPWDTHGDPNDFLLSRHFR

SFIGCPGHGDFESGSYSKANSYSKLSTSPVQTSGYMRYGDDNFSPTSFPELADLASQNIYPTPNSYLPKT

NFLIPSAFQTSPLLQKFIFTEPIFNYSDKVVSMVHEVLYGCRQNINLVQDILHQALLLPLHCHKAISLVI

MVYGSWLENKQNRPIFMQALDTPIKNSDQLLKSVYASQSIEENRAFQINNHLSDFKQIQQSQAGNSPTDK

MKPEDNASDPEELKGCLQNTIQIMLENMANVFYASSLDVSFSGPQNTNRVATNTADYTKHQIDLCRLVLQ

VFQFASNSNELTRETWSKLLSILMDIMSRTMINTSPTNVQNYKWLLNNKLTQNLFQTLNGALLRASLFST

ISSEPWDQCLNVYSKLMHWPSLIIEWKKVMRMLTCTMAKLVYGVDLSDLPQEKKRPRFKRLAASTNSASR

ARPKSLTESALNYNNNNNPNINSSASVLLSASVAYPSALSIANFHQNSLDDNLILNSGCTEPDNNIGDNR

DISRDLSKNMSATCSDVKALQNHDHKPSIEKWTSETAHHETTNTDEMNNMINNDNTRSHSSINYDGVADD

QSNLEECNDDSTVMNNIKHRHNSQSFPHDLLTKTELVQLKADAKDKTLMSERRRLSFVRRATSEVALEVK

FGLDSPESPVRTYFSESQGVNGLDILRSYDRPNNEGTNLQLSRQDTLTATQEVPIANGSSKVATAAANDQ

LNTVDVPRCILAGGPAFGWTNESIVICWRRFLGVLGNFHKITNPTTLSDIFSYLTELTTCLLKIREYQTV

PCSTDTCIQPIPHFVPPIDFISPILFDSMNLSEDFLEAKQIAIRALIDIAVFYHDGYPNLELIARFFHLI

HQISVTKHEKFIFEVVRSCNIRMFVSPLPSTHLLILDFLSNANVVLANPNPGTEIPRSDALSVVLSLICY

PYHFNRLESIDPMCTELKTIVCQDLTSQLIHILTQSVLSDPSAEVRCLAITGLSIFCVTELINSSSKESG

SVSSTGLNSLFFDSIIILLGMMRFQNRIISIVAVDMINMLADYCHLLLLYDVRLSCLVLLSLAWTLYSAW

DNANLNTISSTDKRFFLSLLQAIIEWSMHIPYDQLIKKFENKTESSLPALNLLDTVIEVLCYIASNDTNS

QCLLNSQNSNITQPKYLPSIYFTDPLLEHISTCKQSIDIKLFYTTESELDLSSLTTKYWIGTNSVNTSIE

STYHNEINHPTESVRLAARMTISHLLNHIDQFPVSKHGAQLNSSIQEHHDQLAYSISTQEHYCTNNVDDA

SELTADIFERNNVQIFVLNRSVILTFISLPNVVKSPSQLKNTGDTTADDKFDAALSNELHAELNSAAYSY

LPLSYLNPNIVGKMSSKSGLVTDKSYTRIITRDLSGKYCWDASYLYGSIYDYVERRKDNQLQFDELANAD

MSLMVDSSDLDLDLSRDPPPCPPPRVNPPRPPLTDENTSHIIDTFDRLNDVLRDLALTSPECSINWCTLK

PSEYENVKKLSDKELTARLNMEKMTYDQITAQCQMDDDTVHRKLADVDVLSLYDSYSLQPTRHKYSLKNT

IHSVINNKNPNELESIKQNTSQFYNSRHLLNQLGYVSWRYRSTVDLLQKSPALVRELKHLDNLGSRETHK

FAVFYVGAGQEDKQSILANQTASLEFENFVAGLGWEINLLTHKGFRGGLERSGRAGLSTPYYATSTLEVI

YHVSTRMPSSTHEDLKYKHLGNDEVMIIWNENSRAFRRSVLRTQFGDVLIIISPLLNGLYKVEVRREAQI

GLFGPIVENAILCANVLPGLVRATAINASRAVQATKPGYRRPYEDRASSLQQIISKHTLSTSFEEYTESI

LFPSSKTVHYDHSSGINKATGVKFSLAPHNKQESLHNYAQTSISHLPNNVNTNNDHNPLKHDNLTLTSSI

RQKEIISVPSRQPTSAHSRTSSVSRSRGKLPGYQYAINLNSNPISVISQPPLKTNSPNVHPTENSDNNRM

SNPITSPRLFRGLKTRRHSGSSKIN

**putative tuberin [Schistosoma mansoni]**

**NCBI Reference Sequence: XP_018650501.1**

>XP_018650501.1 putative tuberin [Schistosoma mansoni]

MFRRHRPSDTSAVKSRKKFLSEKESTHKIKHLKILIDSLSDDELQPFFVENSSLIFQVFSDCFFSFEWDV

KLKGSSNCIKELEVVLTVFEKVLLLLPEHIHQRWQHNCIIEVIEDLLYEKNALTIRKRGIRLFLIWYQIL

GLNATSVCHRIFYNLVPEFGPLIAEYQKRENDQRKRSHPHARNLSTDTRGQMMQESKSSNVVAPRERVAL

LTATALGEDESFDDKSLVLLQSLLHFLVSESFKIEWSQLRYEQHLMQLWFLFEKLKHSYLPVIFPCLSPL

YSIYSPSTSSQVNNTVPLTSNILQDYPIHPSRLSYYQLEFACWLATFVYTGPRESSHAISSDGGFDSNFT

IHGSVVGTTGPSFNLAGSFPWDTHGDPNDFLLSRHFRSFIGCPGHGEFESGSYSKANSYAKLSSSPPVQV

PIYLRNGDENFVQEPTNLAASNLSPKLNSYSVKDTAVIPSTVQTSPLPQKFIFTEPFFNYDEKVVSMVHE

VLYGCRQNINLVQDILHQALLLPLECHKALFFIVTVYGSWLENKHNRPIFMQASDSPVKSITPSAKSQVN

ASSIEHSQMLKMNGRMNDLKQIQQSQIEDFPIDETKSKDDVGLNDPEELKGCLQNTIQIMLENMANIFYA

SSLNPNLSESQNTNRISTSGTDYTKYQIDLCRLVLQIFQFASNSNELTSETWSKLLSILMDIMRKTMVNT

SPSNVQNYKWLSNNKLTQNLFQTLNGALLRASLFSTISSEPWDQCLNIYSQLSHWPSLIIEWKKVMRMLT

CTMSKLVYGVDLSDLPQEKKGPRIKRLAASTTDSIELHLSSQENSQKRFAASNELKTLRNDNNTRQCIEK

WTFDTISSKNYVLDNNNDNGNVVHSYIPPRVSSINGTHDESNLQMSSNDLTEVYGNHALHNSRSISHDLS

NQNESKQFNVTVKSETSISESRRMSSVRRATSEITLEVVKNYQFALQAIESSCNSRFSRFTRDDYDCQLH

SELSNHHDIPRLCDILSDEHSTLEPNSKDILTAAPSQEVSITNETSKLPSNTSNDQLNTADLPRCILAGG

SALGWTHESIVICWRRFLGILGSLHKITNPTTMSDIFNYLNEMTSCLLKIRAYQSVPSVTETCVQPIPQF

VPPVNFIAPILFNTMSLSEEFMEAKKIAIRTLCDIVVRSHDGCPDPELIAYFYYLIHQISVTKQENYVFE

VIRSCNMRLFGSSLPSTHLLILDFLSDVNVVLSNPNSGTEIPRSEATSIILSLLCYPYHFNHLESIDPMS

MKAKTILCIDLKSNLVHSLIQAVLSDPSGEVRCLAITGLSIYCVIELVNSFSRESKTGPSVNFKPMDDLF

LESIIILLGMMRFKNRAISIVAVEMINTLAEYCHLLLYRDAKLPSLVLLSLAWTLYSTWDNMDLDSMSST

DKQFFHSLIQSIIEWSMRIPYDQLMKNFENKAELKLLPASNLLDIIIEVLCFIISNNTNSQTSVQNSNYP

QSKILSSMNFTDPLREYINRCKQLIDINLFNAPKSDLDLSTMTAKSWIVTNAENTMIPYHYNDDMNRTME

SVRLAAQMAMSHLLNHLDQFPMSKQCVQLNTSIQERHDQLAHSISSAQKQFTTNNHSNMDDLSELTAEIF

EQNNLQIFVLDRSIILTFLSLPIVKLPVQLKNINDNVSPTTVSSDKDNTELQVVEFNNSTYSYLPLSYLN

SKDVEVDSSKSSLITDKFYTRIITRDLSGKYSWDVSYLYASLVDMKEKQENQQLNEPTNTNLRLVDSDDS

NSHLITRDPPPCPPPRVNPPPLITNSLAPNTIDQLDRLSGVLRDLAITSPECSINWCTSKLCEYENLKNI

SDKEKAACLNMEKMTCDQITAQYQIDENVTHRKLPDIDVLSLYNSHISQPKTGSKNSLKYTICSLINDRK

ISVQELESMKQNTLQFYNSRHLLNQLGYLSWKHRPTVELLQISPALIRELKHLDNLGSRETHKFAIFYVG

AGQEDKQSILSNQTASLEFENFVAGLGWEVDLLKHKGFRGGLECSGRAGLSTPYYATSTLEVIFHVSTRM

PSSTQEDLKYKHLGNDEIMIIWNENSRAFRRSILRTQFGDVLIIISPLLNGLFKVEVRREAQVGLFGPIV

ENAILTANVLPGLVRATAINASRAVQAIKPGYRHPYEDRASSLRQIISKYTLSTCFEEYAASILLPNSKS

VETV

**Ral GTPase-activating protein subunit alpha-1, variant 3 [Schistosoma haematobium]**

**GenBank: KAH9584695.1**

>KAH9584695.1 Ral GTPase-activating protein subunit alpha-1, variant 3 [Schistosoma haematobium]

MFRRHRPSDTSAVKSRKKFLSEKESTHKIKHLKILIDTLPDDELQPFFVENSSLIFLVFSDCFFSLEWDV

KLKGSSNCIKDLEVVLTVFEKVLLLLPEHIHQRWQHNCIIEVIEDLLYEKNALNIRKRGIRLFLIWYQIL

GLNATSVCHRLFYNLVPEFGSLIAEYQKREKCDQLKHNHLQARKFSTDTRGQVMQESRSSNVVAPRERVA

LLTAAALGEDESSDDKSLVLLRALLRFLVSESFKVEWSQLRYEQHLMQLWFLFEKLKHSYLPVIFPCLSP

SYSIYSPSISSQANNTVPLTSNILQDYPIHPSRLSYYQLEFACWLATFVYTGPRESSHAISSDCGFDSNF

TTHGSVVGTTGPSFNLAGSFPWDTHGDPNDFLLSRHFRSFIGCPGHGEFESGSYSKVNSYSKLSSSPPVQ

ASVYLRNGNENFVQEPTNQAAPNLSPKPNSYLLKDTVVIPSTVQTSPLPQKFIFTELFFNYDEKVVSMVH

EVLYGCRQNIDLVQDILHQALLLPLECHKALFFIVTVYGSWLENKHNRPIFMQALDSPMKCINPPVKPQV

NASSTEHSQMLKTNGRMNDPKQIQQSQIEDCPIDVTISKDDVGVNDPEELKGCLQNTIQIMLENMANIFY

ASSLNLNLSESQNTNKISTTGTDYTKYQIDLCRLVLQIFQFASNSNELTSETWSKLLSILMDIMRKTMIN

TSPNNVQNYKWLSNNKLTQNLFQTLNGALLRASLFSTISSESWDQCLNVYSQLSHWPSLIIEWKKVMRML

TCTMSKLVYGVDLSDLPQEKKGPRIKRLAGSTNPATRTRPKSLTEAALNYHNDNTIITTTTNITATTTTA

TTAYNSNSLATVLSSPPAPYASALSVLSIHQNSLDDDNLALNTDSIELHLSSQENSQKQFATYNELKASR

NDNNTRPCIEKWTFDPGSSENYALDNNSNGNVVHSYISSNVSSINGTHDESNSQVLSNDLTEVYGNHALH

SSRSISHELSNQNESNRFNVTVKSEISISGSRRMSSVRRATSEITLEVVKFDLDSPQSPVTTYFDESIDV

GTSNIPRSCDILSDEHSTLEPSRKDTLTTAPSRLVSTTNETSKLPSTASNDQLDAADLPRCILAGGSALG

WTHESVVICWRRFLGILGSLHKIINPTTMNDTFNYLNEMTSCLLKIRAYQIVPSVTETCVQPIPQFVPPV

NFIAPILFNTMSLSEEFIEAKRIAIRTLCDIVIRSHDGCPDPELIAHFYHLIHQISVAKRENYVFEVIRS

CNMRLFGSSLPSTHLLILDFLSDVNIVLSNPNCGTEIPRSEAISIILSLLCYPYHFNHLESIDPMSMKAK

TILCIDLTSKLVRSLIQAVLSDPSGEVRCLAITGLSIYCVIELVNSFSKESGTVSSVSFTSMDNLFFESI

IILLGMMRFQNRAVSIVAVEMINTLAEYCHLLLYRDAKLPSLMLLSLAWTLYSTWDNTNLDSMSLTDKQF

FHSLIQSIIEWSIRIPYDQLIKNFENKSELKLLPASNLLDTIIEVLCFIISNNTNSQTLMNSVQNSNYPQ

SKILSSINFTDPLGEYINIYKQSIDINLFNAPKSDLDLSTMTAKSWIVANAENSMILYHYNDDMNSAMES

VRLAARMAMSHLLNHIDQFPMSKQCVQLNTSIQERHDQLSHSISSAQKQFTTNNHNNMDDLSELTAEIFG

RNNLQIFVLDRSIILTFLSLPIVKLPDQLRNINDNLLPTTVSSDKDNTELQVLEFNNSTYSYLPLSYLNS

NDVEVDSSKSSLVTDKFYTRIITRDLSGKYSWDVSYLYASLVDMKEKQETQQLNELTNTNLRLVDSDNSN

SHLITRDPPPCPPPRVNPPPLTTNRLTPNTIDQLDRLSDVLRDLAITSPECSINWCTSKLCEYENSKNIS

DKEKTACLNMEKVTCDQITAQYQIDENVNHRKLPDIDVLSLYNSHISLPKTGYRNSLKYTIRSLINDKKI

NVQELESMKQNTLQFYNSRHLLNQLGYLSWKHRPTVELLQISPALIRELKHLDHLGSRETHKLAIFYVGA

GQEDKQSILSNQTASLEFENFVAGLGWEVDLLKHKGFRGGLECSGRAGLSTPYYATSTLEVIFHVSTRMP

SSTQEDFKYKHLGNDEIMIIWNENSRAFRRSILRTQFGDVLIIISPLLNGLFKVEVRREAQVGLFGPIVE

NAILTANVLPGLVRATAINASRAVQATKPGYRHPYEDRASSLRQIISKYTLSTCFEEYAESILLPNSKSV

HRNQSNLSFNRGVVNKSGGVKFSSVSPTTQETYHSYSQNSASDLAPNDITNNNNALKYTDDVAQTPSVRQ

RQQTVSSYRATSAHSRTSSVTRAKSMFSRHHIDNLNTKPISVISQPSLKTNSATLHHHNETPDMYKANVF

NSVPSPRLFRGLRSRQHSGSNKIN

**ral GTPase-activating protein subunit alpha-2 isoform X3 [Mus musculus]**

**NCBI Reference Sequence: XP_036017891.1**

>XP_036017891.1 ral GTPase-activating protein subunit alpha-2 isoform X3 [Mus musculus]

MLVRRSSSPAELELKDDLQQAHGRCRQRQTSESTGSDTVVGYSNEAELPVSPWQACEEDPDLSTPTDAVA

DSDARHWLQLSPTDASNLTDSRECLADDCSIIAGGNLTGWHPDSAAVLWRRVLGILGDVNNIQSPKIHAK

VFGYLYELWYKLAKIRDNLAISLDNQSSPSPPLLIPPLRMFASWLFKATTLPNEYKEGKLQAYKLICAMM

TRRQDVLPNSDFLVHFYLVMHLGLTSEDQDVLNTIIKNCSPRFFSLGLPGFSMLVGDFITAAARVLSTDM

LAAPRSEALTLLGSLVCFPNTYQEIPLLQSVPEVSDVVTGAEDVKHYLINILLKNATEEPNECARCIAIC

SLGVWICEELAQSASHPQVKDAINVIGVTLKFPNKIVAQVACDVLQLLVSYWEKLQMFETALPRKMAEIL

VATIAFLLPSAEYSSVETDKKFIVSLLLCLLDWCMALPVSALLHPVSTAVLEELHPSRAPLLDYIYRVLH

CCVCGSSTYTQQSHYTLTLADLSSTDYDPFLPLANVRNSEPIQYHSSADLGNLLTVEEEKKRRSVELIPL

TARMVMAHLVNHLGHYPLSGGPAVLHSLVSENHDNAHVEGTELSSEVFRSPNLQLFVFNDSTLISYLQTP

AEGPAGGTSGGSLSDVRVIVRDISGKYSWDGKVLYGPLEGRLAPNGRNPSFQISGWHHHTCGPQKDLFNG

EEGDDVLDKLLENIGHTSPECLLPSQLNLNEPSPTPCAMNWDQEKAIMEVILRQSAQEDEYVQRCNSDSS

VTVTSQGQPSPVEPRGPFYFCRLLLDDLGMNSWDRRKNFHLLKKNSKLLRELKNLDSRQCRETHKIAVFY

IAEGQEDKCSILANERGSQAYEDFVAGLGWEVDLSTHCGFMGGLQRNGSTGQTAPYYATSTVEVIFHVST

RMPSDSDDSLTKKLRHLGNDEVHIVWSEHSRDYRRGIIPTAFGDVSIIIYPMKNHMFFITITKKPEVPFF

GPLFDGAIVSGKLLPSLICATCINASRAVKCLIPLYQSFYEERALYLEAIIQNHREVMTFEDFAAQVFSP

SPSYSVSGTD

**ral GTPase-activating protein subunit alpha-2 isoform X10 [Homo sapiens]**

**NCBI Reference Sequence: XP_016883465.1**

>XP_016883465.1 ral GTPase-activating protein subunit alpha-2 isoform X10 [Homo sapiens]

MTLMVAWIRANLCVYISRELWDDFLGVLSSLTEWEELINEWANIMDSLTAVLARTVYGVEMTNLPLDKLS

EQKEKKQRGKGCVLDPQKGTTVGRSFSLSWRSHPDVTEPMRFRSATTSGAPGVEKARNIVRQKATAKRSQ

SISNCVHLSEALPATKSVPLLLHTVSALLPGLSYSSCSHRLSEVEECQQSENAPAAGSGHLTVGQQQQVL

RSSSTSDIPEPLCSDSSQGQKAENTQNSSSSEPQPIQENKGHVKREHEGITILVRRSSSPAELDLKDDLQ

QTQGKCRERQKSESTNSDTTLGCTNEAELSMGPWQTCEEDPELNTPTDVVADADARHWLQLSPTDASNLT

DSSECLTDDCSIIAGGSLTGWHPDSAAVLWRRVLGILGDVNNIQSPKIHARVFCYLYELWYKLAKIRDNL

AISLDNQSSPSPPVLIPPLRMFASWLFKAATLPNEYKEGKLQAYRLICAMMTRRQDVLPNSDFLVHFYLV

MHLGLTSEDQDILNTIIRHCPPRFFSLGFPGFSMLVGDFITAAARVLSTDILTAPRSEAVTVLGSLVCFP

NTYQEIPLLQSVPEVNEAITGTEDVKHYLINILLKNATEEPNEYARCIAVCSLGVWICEELAQCTSHPQV

KEAINVIGVTLKFPNKIVAQVACDVLQLLVSYWEKLQMFETSLPRKMAEILVATVAFLLPSAEYSSVETD

KKFIVSLLLCLLDWCMALPVSVLLHPVSTAVLEEQHSARAPLLDYIYRVLHCCVCGSSTYTQQSHYILTL

ADLSSTDYDPFLPLANVKSSEPVQYHSSAELGNLLTVEEEKKRRSLELIPLTARMVMAHLVNHLGHYPLS

GGPAILHSLVSENHDNAHVEGSELSFEVFRSPNLQLFVFNDSTLISYLQTPTEGPVGGSPVGSLSDVRVI

VRDISGKYSWDGKVLYGPLEGCLAPNGRNPSFLISSWHRDTFGPQKDSSQVEEGDDVLDKLLENIGHTSP

ECLLPSQLNLNEPSLTPCGMNYDQEKEIIEVILRQNAQEDEYIQSHNFDSAMKVTSQGQPSPVEPRGPFY

FCRLLLDDLGMNSWDRRKNFHLLKKNSKLLRELKNLDSRQCRETHKIAVFYIAEGQEDKCSILSNERGSQ

AYEDFVAGLGWEVDLSTHCGFMGGLQRNGSTGQTAPYYATSTVEVIFHVSTRMPSDSDDSLTKKLRHLGN

DEVHIVWSEHSRDYRRGIIPTAFGDVSIIIYPMKNHMFFIAITKKPEVPFFGPLFDGAIVSGKLLPSLVC

ATCINASRAVKCLIPLYQSFYEERALYLEAIIQNHREVMTFEDFAAQVFSPSPSYSLSGTD
