# Supplementary figures and images for: Discovery of Schistosoma mekongi circulating proteins and antigens in infected mouse sera
Source: PLoS One. 2022 Oct 13;17(10):e0275992. doi: 10.1371/journal.pone.0275992 (PMC9562170; doi:10.1371/journal.pone.0275992)

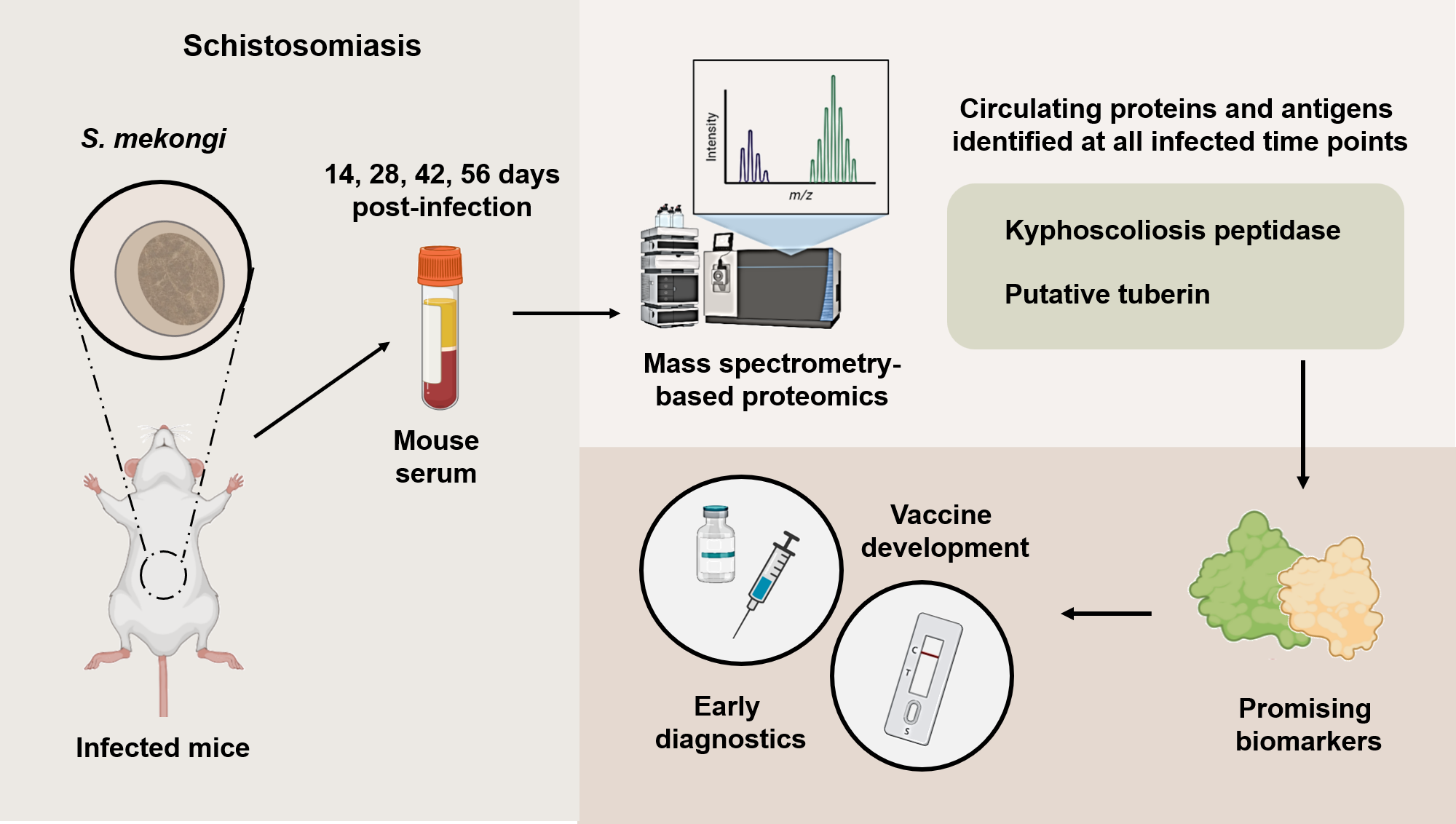

Supplement: S1 Graphical abstract — (TIF) [file pone.0275992.s007.tif]
